# Estrogens regulate early embryonic development of the olfactory sensory system via estrogen-responsive glia

**DOI:** 10.1242/dev.199860

**Published:** 2022-01-13

**Authors:** Aya Takesono, Paula Schirrmacher, Aaron Scott, Jon M. Green, Okhyun Lee, Matthew J. Winter, Tetsuhiro Kudoh, Charles R. Tyler

**Affiliations:** 1 Biosciences, College of Life and Environmental Sciences, University of Exeter, Exeter, Devon EX4 4QD, UK; 2 Department of Biological and Marine Sciences, Faculty of Science and Engineering, University of Hull, Hull HU6 7RX, UK; 3 School of Physiology, Pharmacology & Neuroscience, University of Bristol, Bristol BS8 1TD, UK

**Keywords:** Endocrine disrupting chemicals, Transgenic zebrafish embryo, Embryogenesis, Olfactory glomerular development, Radial glia progenitor cells, Olfactory-mediated behaviour

## Abstract

Estrogens are well-known to regulate development of sexual dimorphism of the brain; however, their role in embryonic brain development prior to sex-differentiation is unclear. Using estrogen biosensor zebrafish models, we found that estrogen activity in the embryonic brain occurs from early neurogenesis specifically in a type of glia in the olfactory bulb (OB), which we name estrogen-responsive olfactory bulb (EROB) cells. In response to estrogen, EROB cells overlay the outermost layer of the OB and interact tightly with olfactory sensory neurons at the olfactory glomeruli. Inhibiting estrogen activity using an estrogen receptor antagonist, ICI182,780 (ICI), and/or EROB cell ablation impedes olfactory glomerular development, including the topological organisation of olfactory glomeruli and inhibitory synaptogenesis in the OB. Furthermore, activation of estrogen signalling inhibits both intrinsic and olfaction-dependent neuronal activity in the OB, whereas ICI or EROB cell ablation results in the opposite effect on neuronal excitability. Altering the estrogen signalling disrupts olfaction-mediated behaviour in later larval stage. We propose that estrogens act on glia to regulate development of OB circuits, thereby modulating the local excitability in the OB and olfaction-mediated behaviour.

## Abstract (precis)

**Summary:** Estrogens regulate olfactory development via estrogen-responsive glia, EROB cells and olfaction-mediated neuronal excitability and behaviour in zebrafish embryos.

## INTRODUCTION

Estrogens are signalling molecules that play crucial roles in the development of reproductive and somatic organs, including the brain. In vertebrates, estrogens are supplied to embryos maternally through the placenta or, in oviparous species, through yolk provisioned into eggs. Estrogens can also be produced locally within the embryonic brain through the conversion of testosterone to estrogens by the enzyme aromatase (Bondesson et al., 2015; Menuet et al., 2005). In rodents, it is thought that the embryonic brain has greater estrogen activity compared with the brain in later life stages, as evidenced by the fact that the highest levels of 17β-estradiol (the most potent endogenous estrogens), aromatase activity and expression of estrogen receptors (ER) (two isoforms of nuclear receptors, ERα and ERβ) occur in the brain at this time (George and Ojeda, 1982; Konkle and McCarthy, 2011; McCarthy, 2008). The importance of estrogen signalling pathways in the developing brain has been further supported by neurodevelopmental phenotypes in ERα and ERβ knockout (KO) mice: ERα contributes to the establishment of sexually dimorphic circuitry and sex-specific behaviour (Ogawa et al., 1998a,b; Simerly et al., 1997; Stephens et al., 2016); ERβ plays crucial roles in cortex development, neurogenesis of calretinin^+^ GABAergic interneurons in the prenatal period (Fan et al., 2006; Wang et al., 2001, 2003) and neural specification of pluripotent stem cells *in vitro* (Varshney et al., 2017). The highest level of ER-mediated transcriptional activation occurs in the neural ectodermal tissues from E12 in the estrogen-responsive element (ERE)-Luciferase mouse model (Della Torre et al., 2018) further evidencing the importance of estrogen signalling in brain development.

However, there is little understanding of how estrogens exert their effects in the embryonic brain before the period of sex differentiation, what the physiological effects of estrogens are or what the developmental consequences are for alterations to estrogen signalling in the brain during embryogenesis. This understanding has a further level of importance given that early-life exposure to environmental contaminants – so-called endocrine disrupting chemicals (EDCs) – that mimic estrogens are thought to cause a wide range of adverse impacts on brain development and function (Derouiche et al., 2015; Kinch et al., 2015; Lichtensteiger et al., 2015; Porseryd et al., 2017), which in turn may lead to defects in cognition, learning, emotional control and behaviour in later life (Gore and Crews, 2009; Braun et al., 2009; Derouiche et al., 2015; Gioiosa et al., 2013; Kinch et al., 2015). In fact, emerging evidence suggests that EDC pollutants in the environment have impacted natural habitats and affected social and reproductive behaviours in wildlife populations (Godfray et al., 2019; Söffker and Tyler, 2012). EDC-induced alterations of estrogen functions during brain development have also been implicated in the pathogenesis of some sex-biased neurodevelopmental diseases in humans, including attention-deficit hyperactivity disorder (ADHD), autism spectrum disorders and schizophrenia (Crider and Pillai, 2017; Mustieles et al., 2015). Thus, a suitable model to study the contributions of estrogens to embryonic brain development in real-time has been highly sought after.

In this work, combining the use of estrogen biosensor (Green et al., 2016; Lee et al., 2012a), calcium sensor (Winter et al., 2021, 2017) and chemical/genetic cell ablation zebrafish models, we reveal a new physiological function of estrogens in the early developing brain which commences shortly after the initiation of neurogenesis. Our findings demonstrate for the first time that estrogens exert a highly specific effect on the olfactory bulb (OB) of the embryonic brain and contribute to the development and function of the olfactory sensory system through newly identified target glia. Given that this occurs shortly after the regional specification of the embryonic brain, at a much earlier stage [24 h postfertilisation (hpf) to 96 hpf] than the initiation of gonadal sex differentiation [i.e. 20-25 days postfertilisation (dpf)] (Lau et al., 2016; Uchida et al., 2002) and/or sex dimorphism in the brain (i.e. 20-40 dpf) (Lee et al., 2018) in zebrafish, this estrogen-mediated cascade is likely a fundamental mechanism required for development of the olfactory sensory system, regardless of sex.

## RESULTS

### The estrogens/ER-mediated transcriptional activation occurs in the OB in the zebrafish embryonic brain

**Fig. 1.**
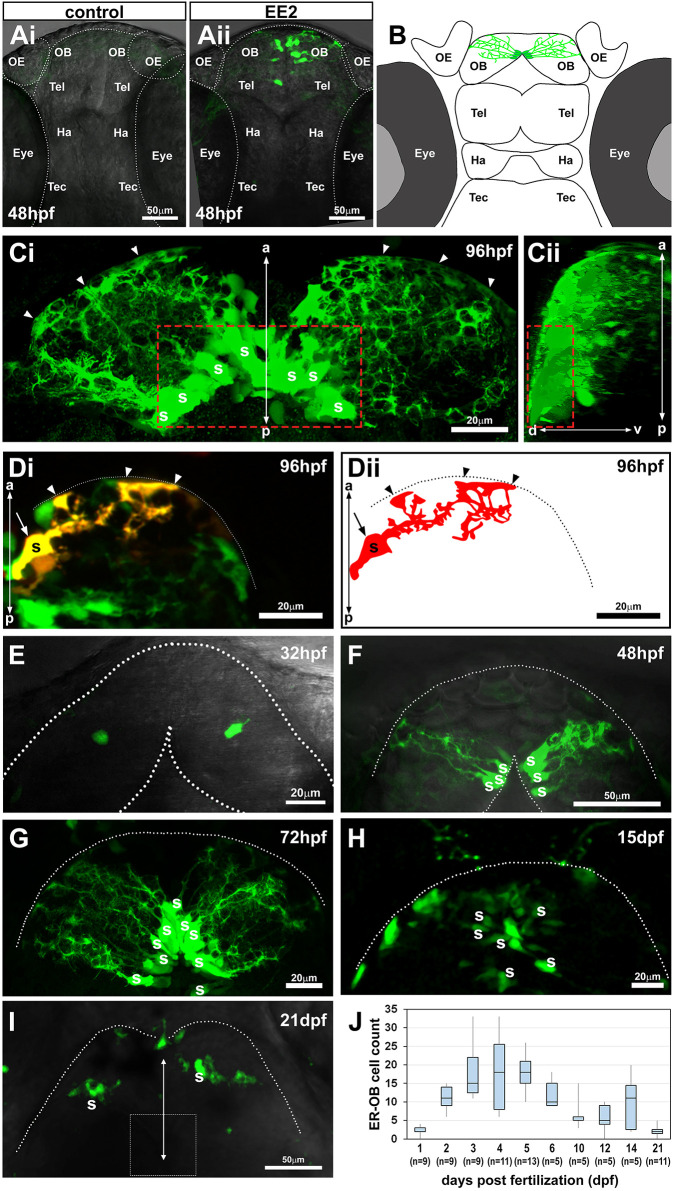
**The earliest estrogens/ER-mediated transcriptional activation occurs in a small number of cells in the OB in the zebrafish embryonic brain.** (Ai,Aii) Confocal *z*-projection images of control (Ai) or 17-α ethinylestradiol (EE2, 100 ng/l; Aii)-exposed zebrafish ERE:GFP embryos at 48 hpf. (B) Illustration of the EROB cellular domain in a 4 dpf zebrafish embryo. (Ci,Cii) Confocal *z*-projection images (Ci, dorsal view; Cii, a 90° rotated view) of EROB cells in EE2-exposed ERE:GFP embryos at 96 hpf. Red dotted rectangles outline the mediodorsal OB; arrowheads show the distal ends of EROB cells. (Di,Dii) Di shows the morphology of a single EROB cell from a confocal *z*-projection image and Dii illustrates a trace of the cell morphology. The midline is on the left edge; dotted lines show the OB pia; arrowheads show the termini of EROB cells at the OB pia; arrow shows the somata of the EROB cell. (E-I) Confocal *z*-projection images of EROB cells (green) showing their ontological development in the brain of embryo/larval zebrafish (dorsal view). Each animal stage was exposed to 100 ng EE2/l for up to 4 days before the indicated developmental stage. Dotted lines show the OB pia; white dotted square in I shows the mediodorsal OB. (J) Ontogenic profiles of EROB cell numbers. Boxplot shows median values (middle black bars) and 1st-3rd interquartile ranges (boxes); whiskers extend to the minimum and maximum of the data range within 1.5× the interquartile range. a-p, anterior-posterior axis; d-v, dorso-ventral axis; OB, olfactory bulb; OE, olfactory epithelia; Ha, habenula; S, somata; Tec, tectum; Tel, telencephalon.

To understand the role of estrogens in embryonic brain development, we used an estrogen biosensor zebrafish model (ERE:GFP) which allows the identification of *in vivo* cell responses through estrogens/ER-mediated induction of GFP (Green et al., 2016; Lee et al., 2012a,b). In ERE:GFP embryos exposed to ethinylestradiol (EE2), a synthetic derivative of the female sex hormone 17β-estradiol, we observed that estrogen response (indicated by GFP expression) occurred primarily in the OB located in the anterior-most region of the forebrain (Fig. 1Ai, control; Fig. 1Aii, EE2-exposed sample), where olfactory signals are first processed in the brain (Miyasaka et al., 2013). These GFP-positive cells were located around the midline ventricular region of the anterior forebrain. By 96 hpf the number of these estrogen-responsive cells in the OB (EROB cells) was much increased (Fig. 1B,Ci), with their projections extending laterally towards the pia of the OB (Fig. 1Ci,Cii). The unique morphology and location of EROB cells were further confirmed by injecting UAS-DsRed DNA into ERE:GFP embryos, which renders a mosaic expression of DsRed in a subset of the EROB cells (Fig. 1Di,Dii). We next investigated the ontogeny of the EROB cells during embryonic and larval brain development. To do this, ERE:GFP embryos were exposed to EE2 for up to 96 h (4 days) before imaging at successive stages of development. We detected the EROB cells with the cellular projections starting to develop after 32 hpf (Fig. 1E) and with complex projection networks formed by 48 hpf (Fig. 1F). The number of EROB cells greatly increased between 48 hpf and 72 hpf (Fig. 1G), reaching a maximum number at around 4-5 dpf (Fig. 1J) and then gradually decreased thereafter (Fig. 1H,I,J). In later life stage (15 and 21 dpf), the size (length) and number of EROB cell projections were seen to be markedly reduced and the somata now dispersed from their original position at the mediodorsal OB (Fig. 1H,I). The localisation of the EROB cells and their ontogenic profiles suggest they have a role in olfactory development in early embryo-larval brain development.

### EROB cells are aromatase B-positive glia

**Fig. 2.**
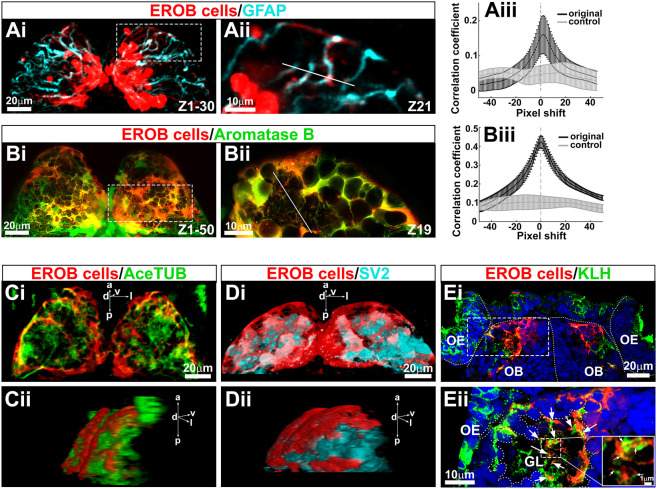
**EROB cells are GFAP- and aromatase B-expressing glia that interact with olfactory sensory neurons at olfactory glomeruli.** (Ai) Confocal *z*-projection images of EROB cells (mCherry, red) and GFAP (cyan) in an EE2-exposed 4 dpf ERE:mCherry embryo. (Aii) An optical section image (1.5 µm step size) magnified from the area marked with white dotted rectangle in Ai. (Aiii) Pearson's correlation coefficient in two dimensions for EROB cells versus GFAP, plotted against image displacement in the *x*-direction (black). Control corresponds to image sets with one channel rotated by 90° (light grey). The peak correlation coefficient (PCC) was found at a 1.5 pixel shift (0.22 µm/pixel size), suggesting a close localisation of EROB cell and GFAP in the same projection (data are mean±s.e.m.). (Bi) Confocal *z*-projection images of 4 dpf EE2-exposed double transgenic – Tg(*ERE:mCherry*)×Tg(*cyp19a1b:GFP*) – embryo. (Bii) One optical section image (0.7 µm step size) magnified from the area marked with white dotted rectangle in Bi. (Biii) Pearson's correlation coefficient in two dimensions for EROB cells versus aromatase B. The PCC was at 0 pixel shift, indicating co-localisation of EROB cell and aromatase B. *P<0.05 (*t*-test for significant difference from the rotated control image sets, grey). Data are mean±s.e.m. (*n*=3). (Ci,Cii) A 3D image of EROB cells (red) and acetylated tubulin-positive axonal projections (green) in the OB (Ci, dorsal view; Cii, a 50° rotated view of Ci). (Di,Dii) A 3D image of EROB cells (red) and olfactory glomeruli stained with SV2 antibody (cyan) (Di, dorsal view; Dii, a 45° rotated view of Di). (Ei) Frontal cryosection image of EROB cells (red), OSNs (KLH, green) and nuclei (Hoechst, blue). White dotted lines indicate the outline of the olfactory epithelia (OE), olfactory bulb (OB) and midline. (Eii) A magnified image of a glomerulus (GL) from the area indicated with a white dotted rectangle in Ei. Arrows indicate EROB cell–KLH^+^ OSN interactions. Outline of the GL is indicated with white dotted freeform line. Magnified image of a central area of the GL is inserted on the right, showing that EROB cells (red) tightly intermingle with OSNs (green) within the GL (indicated with white arrows). a, anterior; d, dorsal; l, lateral; p, posterior; v, ventral.

The morphology of the EROB cells and their location in the OB suggest they may be glial cells. In fact, we found that the projections from these EROB cells labelled strongly with anti-Glial Fibrillary Acidic Protein (GFAP) antibody, a marker for astrocytic glial cells (Fig. 2Ai,Aii). The EROB cells also expressed the brain-specific estrogen synthesising enzyme aromatase B gene (*cyp19a1b*) as shown by the overlap with *cyp19a1b:GFP* (Brion et al., 2012) in double transgenic embryos carrying both *ERE:mCherry* and *cyp19a1b:GFP* reporter genes (Fig. 2Bi,Bii). Co-localisation of EROB cells and GFAP in the same projection (Fig. 2Aiii) and co-localisation of EROB cells and aromatase B expression (Fig. 2Biii) were confirmed using a method based on Pearson's correlation coefficient in two dimensions (Steinfeld et al., 2015). We also found that EROB cells co-expressed Sox2 (a marker of neural progenitor cells) (Fig. S1Ai-Aiv,Bi,Bii), but they were segregated from the brain region in which Elavl3 (HuC)-positive immature post-mitotic neurons were localised (Fig. S1Ci-Civ,Di,Dii). These data demonstrate that EROB cells are not neurons but Sox2^+^/GFAP^+^/aromatase B^+^ expressing glia.

### EROB cells interact with olfactory sensory neurons at olfactory glomeruli via their projections

We next examined for connectivity between EROB cells and neurons in the OB by co-staining EROB cells (mCherry) with antibodies against anti-acetylated tubulin, a marker for axonal projections, and against anti-synaptic vesicle glycoprotein 2 (SV2), a marker for synaptic neuropils, including olfactory glomeruli. We confirmed that the projections of the EROB cells spanned the entire surface of the OB (Fig. 2Ci,Cii) and overlaid olfactory glomeruli (Fig. 2Di,Dii). The distal ends of EROB cell projections were associated closely with the acetylated tubulin^+^ axons at olfactory glomeruli (Fig. S2A,Bi-Biv) and surrounded each SV2^+^ glomerulus (Fig. S2C,Di-Div). The interaction between EROB cells and olfactory sensory neurons (OSNs) was further examined by co-labelling EROB cells (mCherry) with an antibody against anti-keyhole limpet haemocyanin (KLH), a specific marker for OSNs, in frontal cryosections of the OB. This showed that the EROB cells projected to the glomerulus at the point where they connected with the axonal termini of KLH^+^ OSNs (Fig. 2Ei,Eii). These projections from EROB cells intricately intertwined with KLH^+^ OSNs in the olfactory glomeruli (Fig. 2Eii, high magnification image). These data suggest that EROB cells may act as cellular scaffolds for supporting the axonal growth of OSNs to specific glomeruli in the OB and for winding the termini of OSNs into a spherical shape within a glomerulus.

### Estrogens regulate EROB cell projections

**Fig. 3.**
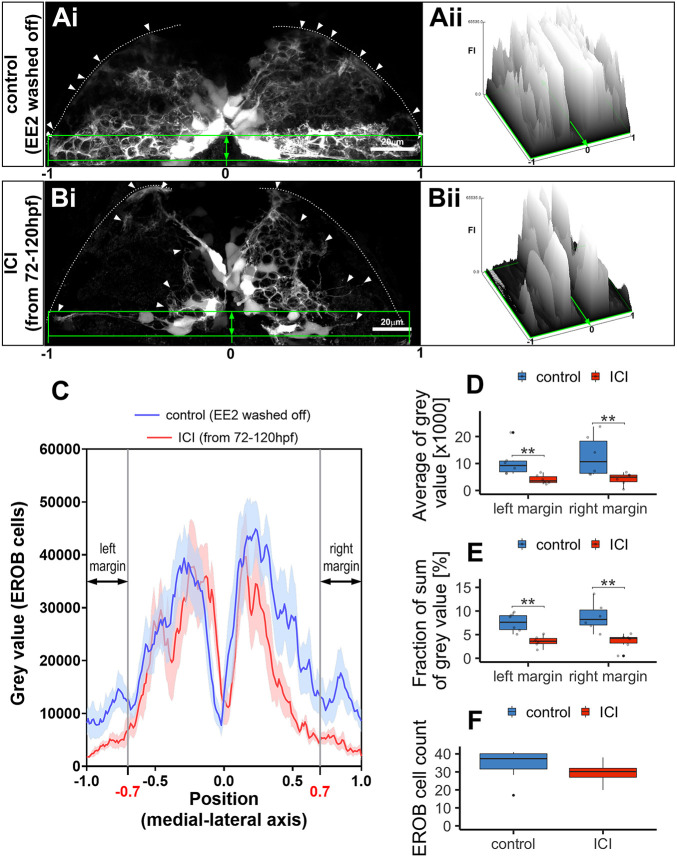
**Estrogens regulate EROB cell projections.** (Ai,Bi) Confocal *z*-projection image of EROB cells in EE2-exposed (from 1 hpf to 72 hpf, then washed off) control (Ai) or subsequently an ER antagonist, ICI 182,780 (ICI, 10 µM)-treated (from 72 hpf to 120 hpf) (Bi) 5 dpf ERE:GFP embryo (see details in Materials and Methods). Arrowheads show the distal end of the EROB cells; white dotted line indicates the pia of the OB; green rectangles highlight EROB cell margins. (Aii,Bii) Surface plots of fluorescence intensity (FI) of GFP signals (EROB cells) in the EROB cell margins in control (Ai) and ICI-treated (Bi) embryos. The surface plots are shown along the width of the EROB cell margin, adjusted by the position of the midline as ‘0’ and the positions of lateral ends as ‘−1 (left)’ or ‘1 (right)’. (C) The intensity plots of GFP signals (EROB cells) in the EROB cell margin in control (blue) and ICI (red) embryos. *X*-axis is adjusted as described above. Data are mean±s.e.m., *n*=6 each. Black double arrows indicate both edges of the EROB cell margin, at the positions of [−1:−0.7] (left margin) and [0.7:1] (right margin). (D,E) The average of grey value and sum of grey value (percentage of total grey value in each embryo) of left and right margin (*n*=6 each). **P<0.01 (Mann–Whitney *U*-test). (F) The total EROB cell count (*n*=8, *P*=0.19, *t*-test). Boxplots show the median (middle black bar) with 1st and 3rd quartiles of the distribution. Whiskers extend to the minimum and maximum of the data range within 1.5× the interquartile range; data beyond that range are defined as outliers and plotted individually in black. Overlaying the boxplot is a scatterplot in semi-transparent showing all individual observations.

We then questioned whether estrogen activity is required for the establishment of EROB cell projections, which appear to be an integral structural component of the OB. To address this, ERE:GFP embryos were treated with EE2 from 1 hpf to 72 hpf to induce GFP in EROB cells and then underwent either a washout period between 72 hpf and 120 hpf [referred as ‘control (EE2 washed off)’] or subsequent treatment with an ER antagonist (ICI182,780, ICI) to inhibit estrogen activity between 72 hpf to 120 hpf [‘ICI (from 72 to 120 hpf)’]. The control group showed intense EROB cell projections overlaying the majority of OB surface (Fig. 3Ai,Aii). In contrast, the ICI-treated group showed substantial alteration to the EROB cell morphology (Fig. 3Bi,Bii). To quantify this effect of the ICI, mean GFP intensity within the EROB cell margin (marked with a green rectangle in Fig. 3Ai or 3Bi) was plotted along the width of the OB margin. The ICI-treated group showed a consistent reduction in GFP signals near left and/or right lateral ends of the OB compared with the control group, representing distorted EROB projections in ICI groups (Fig. 3C-E). This ICI effect was unlikely to be due to an effect on EROB cell number as the EROB cell count did not differ between the control and ICI group (Fig. 3F). These data indicate that estrogen activity is essential for the establishment of EROB cell projection networks during OB development.

### Estrogens/EROB cells are essential for olfactory glomerular development

**Fig. 4.**
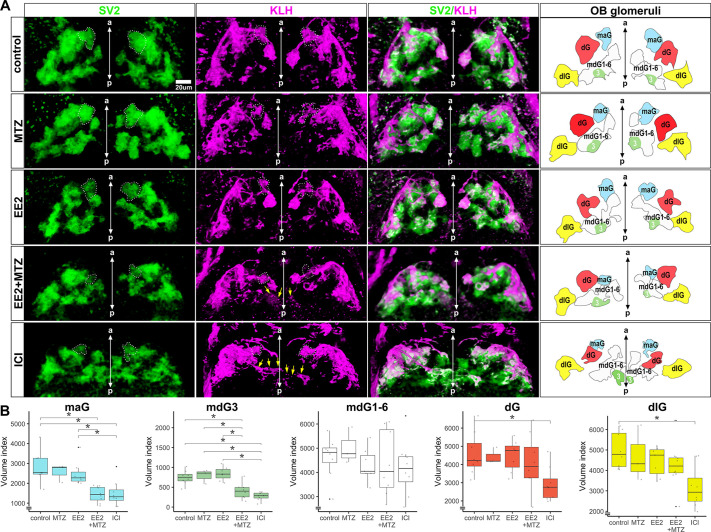
**EROB cell ablation impairs the development of olfactory glomeruli.** (A) Representative 3D images of olfactory glomeruli (SV2, green), OSNs (KLH, magenta) and merged image of SV2/KLM in control, MTZ, EE2, EE2+MTZ or ICI-exposed 4 dpf ERE:mCherry embryos. Right-end panels are the illustrations of OB glomeruli map generated based on the outlines and positions of five selected olfactory glomeruli in the representative 3D images: maG, blue; dG, red; dlG, yellow; mdG1-6, white; mdG3, green. The midlines are indicated with anterior-posterior (a-p) axis in the images. White dotted lines in SV2 and KLH images show maG; green coloured areas in OB glomeruli map show mdG3; yellow arrows in KLH images show altered axonal extensions of OSNs. (B) Relative volumes of the five different selected glomeruli. *n*=8 for control, EE2, EE2+MTZ and ICI, *n*=3 for MTZ alone. Data are mean±s.e.m. shown. *P<0.05 (ANOVA with Tukey post-hoc test). Boxplots show the median with 1st and 3rd quartiles of the distribution. Whiskers extend to the minimum and maximum of the data range within 1.5× the interquartile range; data beyond that range are defined as outliers and plotted individually in black. Overlaying the boxplot is a scatterplot in semi-transparent showing all individual observations.

To assess the inter-relationship between estrogens and the EROB cells in the development of the OB we blocked estrogen signalling through the application of ICI or ablated the EROB cells using nitroreductase-mediated chemical/genetic cell ablation (Curado et al., 2008) (details in Materials and Methods, Chemical exposure). EROB cell ablation was optimised using a combined exposure to EE2 and a prodrug metronidazole (MTZ) from 1 hpf to 33 hpf, which leads to an effective EROB cell ablation in the absence of developmental abnormalities (Fig. S3A,B). In this work we measured the locations and volumes of five different olfactory glomeruli – medioanterior glomerulus (maG), dorsal glomerulus (dG), dorsolateral glomerulus (dlG), mediodorsal glomerulus 3 (mdG3) and a group of mediodorsal glomeruli (mdG1-6). These were selected because they are located in close proximity to the dorsal surface of the OB (Braubach et al., 2013) and apparently interacting directly with EROB cells. In addition, we examined the axonal extension patterns of OSNs projecting towards olfactory glomeruli. EROB cell ablation (EE2+MTZ) and pharmacological inhibition of estrogen signalling (via ICI) altered the stereotypical positions and volumes of some of OB glomeruli: with EE2+MTZ or ICI, mdG1-6 were often ectopically positioned, for example being closer to the midline compared with the control condition (Fig. 4A, SV2 images and illustration of OB glomeruli map). In addition, mdG3 was much smaller in size or was absent in EE2+MTZ- and ICI-exposed embryos (Fig. 4A). Of the five examined glomeruli, maG and mdG3 were the most severely affected by the EE2+MTZ or ICI treatments, leading to reductions in their volumes (Fig. 4B, maG and mdG3). We also observed atypical axonal projections of OSNs in EE2+MTZ- and ICI-treated embryos, with their projections now extended towards the midline of the OB (Fig. 4A, yellow arrows in KLH images). These phenotypes were not seen in control samples or in embryos treated with EE2. These data support that estrogens and EROB cells are crucially involved in olfactory glomerular development.

### Estrogens promote inhibitory synapse formation in the OB

**Fig. 5.**
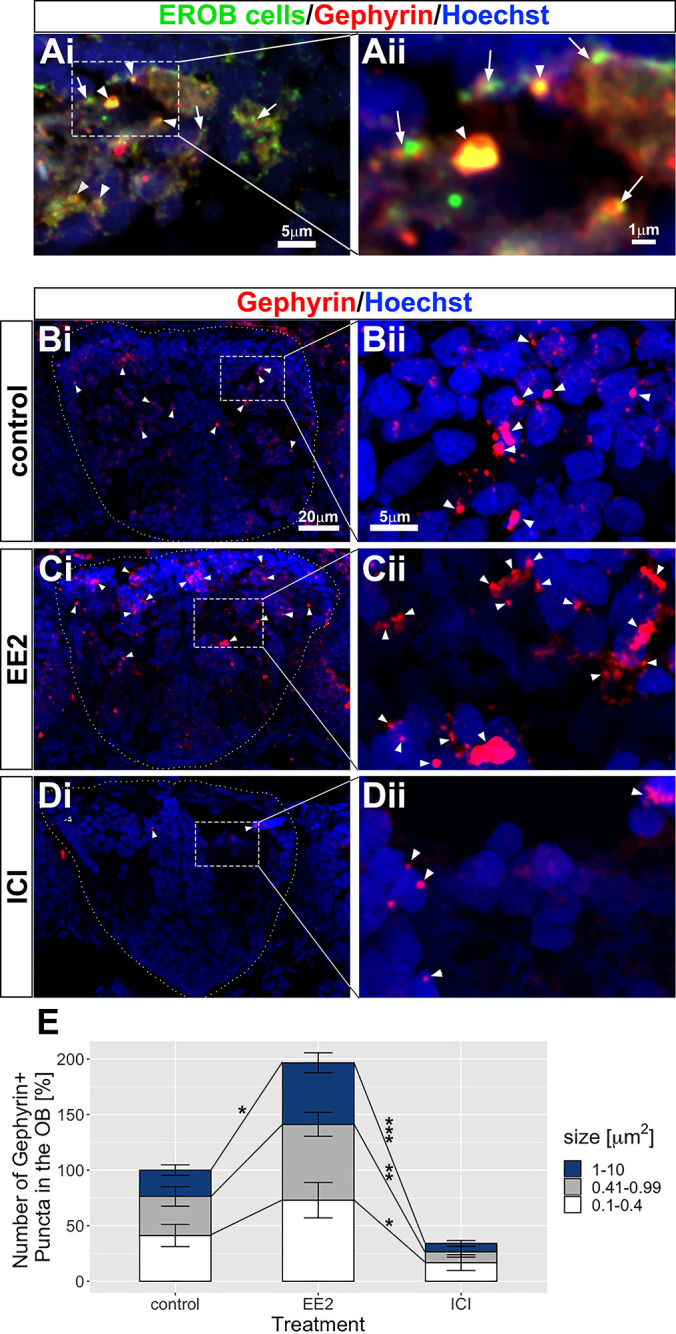
**Estrogens promote inhibitory synaptogenesis in the OB.** (Ai,Aii) A transverse cryosection image of EROB cells (green), gephyrin puncta (red) and nuclei (blue) in EE2-exposed 4 dpf ERE:GFP embryos. The distal-ends of EROB cell projections co-localise with (arrowheads) or localise in close proximity to (arrows) gephyrin+ inhibitory synapses at an olfactory glomerulus. Aii shows a magnified image of a sub-region of olfactory glomerulus in Ai. (Bi-Dii) Estrogens promote inhibitory synaptogenesis in the OB, whereas ICI inhibits the formation of inhibitory synapses. (Bi,Bii) control; (Ci,Cii) EE2-treated; (Di,Dii) ICI-treated 4 dpf ERE:GFP embryos. Gephyrin puncta (red) in the OB are shown with arrowheads. Bii, Cii and Dii show magnified images of a sub-region of the OB indicated in Bi, Ci and Di, respectively. (E) Percentage increase in the total numbers of gephyrin+ puncta in the OB. The number of gephyrin+ puncta was quantified for three different sizes, 0.1-0.4 µm^2^ (white), 0.41-0.99 µm^2^ (grey) and 1-10 µm^2^ (blue). Data are mean±s.e.m., *n*=4. *P<0.05, **P<0.01, ***P<0.001 (ANOVA with Tukey post-hoc test).

Our findings show that estrogen signalling and its target EROB cells play a key instructive role in olfactory glomerular development. It is known that inhibitory regulatory circuits at olfactory glomeruli, which comprise various types of inhibitory interneurons, are consecutively established during the course of olfactory glomerular development (Batista-Brito et al., 2008; Nagayama et al., 2014). We therefore examined whether estrogen signalling and EROB cells could also regulate the local synapse formation in the OB. To address this, firstly we co-stained EROB cells (GFP) with gephyrin, a postsynaptic scaffolding molecule for inhibitory synapses (Tyagarajan and Fritschy, 2014) to analyse the spatial relationship between EROB cells and gephyrin+ inhibitory synapses in the OB. As shown in Fig. 5Ai,Aii, EROB cell projections were located closely with gephyrin-expressing puncta at olfactory glomeruli. We found that the EE2 treatment increased the number of gephyrin+ puncta in the OB (Fig. 5Ci,Cii), whereas inhibiting estrogen signalling using ICI markedly reduced the number of puncta (Fig. 5Di,Dii) compared with those in the control (Fig. 5Bi,Bii). Notably, EE2 increased both the number of gephyrin+ puncta and also the puncta size. This effect of EE2 was greater in the larger-sized puncta (Fig. 5E). Conversely, when estrogen activity was inhibited by ICI, the number of gephyrin puncta was greatly reduced (Fig. 5E), indicating that estrogen signalling is essential for establishing inhibitory synapses in the OB. These data suggest that estrogens regulate inhibitory synaptogenesis.

### Estrogen signalling and EROB cells specifically modulate the intrinsic/spontaneous excitability in the OB in the embryonic brain

**Fig. 6.**
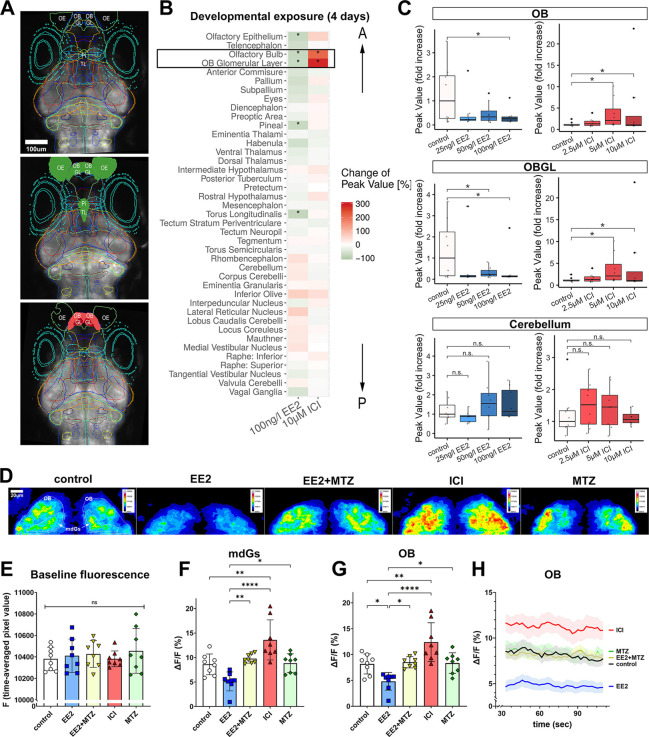
**Estrogens/EROB cell cascade inhibits the intrinsic spontaneous neuronal activity in the OB.** (A) LSM images of 4 dpf *elavl3*:*GCaMP6s* zebrafish embryo brain (dorsal view) of control (top), EE2-exposed (middle) and ICI-exposed embryos (bottom). Each coloured line represents a brain ROI. ROIs for which GCaMP activity is inhibited by EE2 are coloured with green and those activated by ICI are coloured with red (based on the data in Fig. 6B). (B) Heat map displaying changes (%) of GCaMP signals in 41 different brain regions. A black rectangle indicates the most affected ROIs (OB and OBGL). The order of ROIs represents the location of a ROI along anterior-posterior (A-P) axis (top-bottom). *P<0.05 (likelihood ratio test with Tukey's post-hoc test), *n*=8 each. (C) Dose-dependent effects of EE2 and ICI on intrinsic neuronal activity. *P<0.05 (likelihood ratio test with Tukey's post-hoc), *n*=8 each. Boxplots show the median with 1st and 3rd quartiles of the distribution. Whiskers extend to the minimum and maximum of the data range within 1.5× the interquartile range; data beyond that range are defined as outliers and plotted individually in black. Overlaying the boxplot is a scatterplot in semi-transparent showing all individual observations. (D) Representative confocal *z*-projection images of time-averaged GCaMP6s signals in the OB of control, EE2-, ICI-, MTZ- and EE2+MTZ-exposed 4 dpf Tg(*ERE:mCherry*)×Tg(*elavl3:GCaMP6s*) embryos. Representative ROIs of left and right mdG1-6 (mdGs) and ROIs of left and right OB are marked with white lines in the control image. (E) Raw baseline fluorescence signal (F) (time-averaged pixel value) of experimental groups. (F,G) Time-averaged intrinsic neuronal activity in mdGs (F) and in OB (G) from each experimental group (*n*=7-9) are shown as ΔF/F (%). *P≤0.05, **P≤0.01, ****P≤0.0001 (ANOVA with Tukey post-hoc test). (H) Time-course of the intrinsic neuronal activity in the OB. n.s., not significant; OB, olfactory bulb; OBGL, olfactory bulb glomerular layer; OE, olfactory epithelia; Pi, pineal; TL, torus longitudinalis.

Given that the estrogens/EROB cell cascade crucially and selectively regulates olfactory glomerular development, we examined whether alteration of estrogen signalling affects local neuronal activity in the OB in the developing embryos. To do that, we employed a calcium sensor *elavl3(huC):GCaMP6s* (hereafter *elavl3:GCaMP6s*) transgenic zebrafish model in combination with light sheet microscopy (LSM) and a GCaMP6s image processing pipeline established previously in our laboratory (details in Materials and Methods) (Winter et al., 2021, 2017). Using this system, a full brain volume of GCaMP6s images can be captured that allows us to extract region of interest (ROI)-specific neuronal activity data. We found that developmental exposure to EE2 (from 1 hpf to 4 dpf) most significantly reduced the intrinsic neuronal activity in the OB and OB glomerular layer (OBGL) out of 41 different ROIs compared with those in the control condition (Fig. 6A, EE2, highlighted in green; Fig. 6B, marked with black rectangle). Conversely, developmental exposure to ICI specifically increased GCaMP signals in the OB and the OBGL. (Fig. 6A, ICI, highlighted in red; Fig. 6B). Dose-response experiments confirmed that the effects of EE2 and ICI on the intrinsic excitability were inverse, dose-dependent and specifically observed in the OB and OBGL but not in other brain regions, such as the cerebellum (Fig. 6C). To clarify whether this OB-specific change in excitability was due to the acute response to the estrogen signalling pathway (Kramar et al., 2009; Zhang et al., 2010), or was in fact a long-term response, we examined the effect of transient exposure to EE2 or ICI on the intrinsic neuronal activity in the same assay system. Unlike the developmental exposure to EE2, transient EE2 exposure (20 min before recording) did not cause changes in excitability in the OB (Fig. S4). Furthermore, the impact of transient exposures of ICI were not specific to the OB and were more widespread (albeit more mild responses; Fig. S4). These results suggest that estrogen signalling regulates the intrinsic excitability in the embryonic brain through a long-term action of estrogen signalling rather than by a transient regulation of the neuronal activity. Next, we investigated whether EROB cells are involved in estrogen-mediated inhibition of excitability in the OB and, if so, whether ablation of EROB cells could prevent this estrogen-mediated effect. To assess this, we used Tg(*ERE:nfsBmCherry*)×Tg(*elavl3:GCaMP6s*) embryos that allow a simultaneous analysis of both nitroreductase-mediated chemical/genetic EROB cell ablation and live GCaMP6s imaging in the same embryo (Fig. S3C). Owing to the heterozygosity of *elavl3:GCaMP6s*, the Ca^++^ signal was reduced to 50% using this transgenic line. We therefore used a Zeiss 880 Airyscan ‘fast-mode’ to acquire the volumetric GCaMP signals in the OB over ∼2 min (details in Materials and Methods). In EE2+MTZ-exposed (EROB cell ablated) embryos, the level of Ca^++^ signals in the OB and/or in mdGs were restored to a level comparable with that observed in control embryos (Fig. 6D). Quantifications of the time-averaged dF/F (%) of GCaMP signals revealed that EE2 exposure inhibited the intrinsic excitability both in specific glomeruli (mdGs) (Fig. 6F) and in the entire OB (Fig. 6G). In contrast, ICI conversely activated the intrinsic neuronal activity, and EROB cell ablation by EE2+MTZ treatment prevented such EE2-induced neuronal inhibition (Fig. 6F,G). We confirmed that baseline signal F remained constant in all experimental groups (Fig. 6E) and there was no significant time-window deviation in dF/F (%) in all treatment conditions (Fig. 6H). These data suggest that estrogens consistently inhibit the intrinsic neuronal activity, and this is likely mediated via EROB cells.

### Estrogens inhibit olfaction-evoked neuronal activation via EROB cells and abolish olfaction-mediated avoidance behaviour

**Fig. 7.**
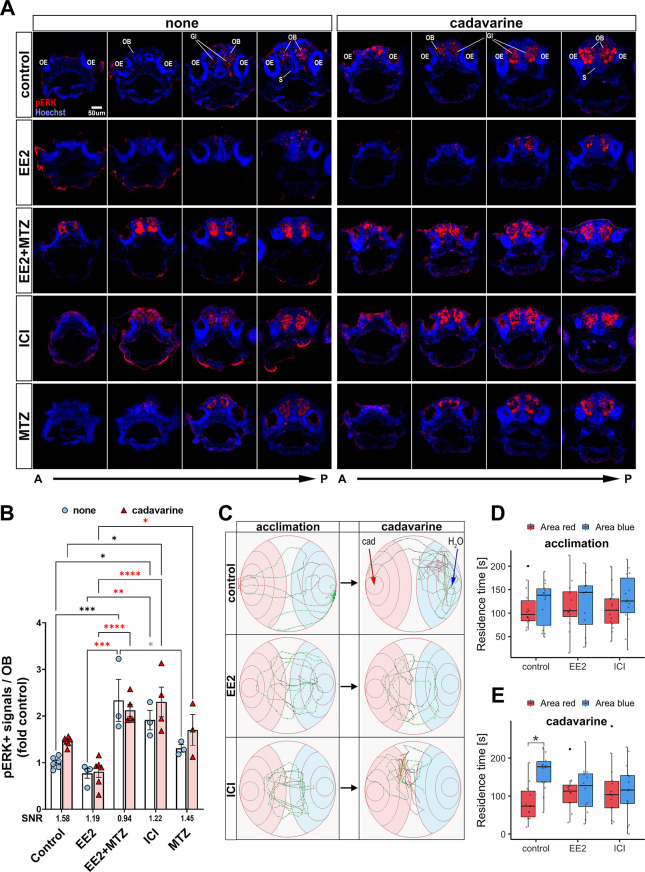
**Estrogens inhibit olfaction-evoked neuronal activation and abolish olfaction-mediated avoidance behaviour.** (A) Representative sequential forebrain transverse section images of pERK- (red) and nuclei (blue)-stained none (left) and cadavarine-stimulated (right) control, EE2-, EE2+MTZ-, ICI- or MTZ-treated 5 dpf ERE:mCherry larvae. (B) pERK signals within ∼50 µm A-P volume of the OB of none (white bar/pale blue dots) or cadavarine-stimulated (red bar/red triangles) 5 dpf ERE:GFP larvae (*n*=3-6) are shown. *P≤0.05, **P≤0.01, ***P≤0.001, ****P≤0.0001 (ANOVA with Tukey post-hoc test). Black, red and grey asterisks show comparisons with control, EE2 and EE2+MTZ, respectively. Signal/noise ratio (SNR) was calculated as ‘the mean of the odour-evoked pERK levels/the mean of the basal pERK levels’ (shown below the *x*-axis). (C) Representative swimming trajectories of control (top row), EE2- (middle row) and ICI-(bottom row) exposed 9-11 dpf ERE:mCherry larvae. Trajectories (5 min) during acclimation (left column) and after cadavarine administration (right column) from the same larva are shown. The administration sites of either cadavarine (red arrow) or water (blue arrow) are indicated in control/cadavarine-stimulated trajectory (top/right). (D,E) Averaged residence time in red (cadavarine) or blue (water) area during acclimation (D) and after cadavarine administration (E) in control (*n*=14), EE2- (*n*=12) or ICI- (*n*=12) treated larvae. *P<0.05 (likelihood ratio test with Tukey's post-hoc test). Boxplots show the median with 1st and 3rd quartiles of the distribution. Whiskers extend to the minimum and maximum of the data range within 1.5× the interquartile range; data beyond that range are defined as outliers and plotted individually in black. Overlaying the boxplot is a scatterplot in semi-transparent showing all individual observations. A-P, anterior-posterior axis; Gl, olfactory bulb glomerular layer; OB, olfactory bulb; OE, olfactory epithelia; S, subpallium.

Next we tested whether EE2, ICI or EROB cell ablation alter odour-evoked neuronal activation (phosphorylation of ERK/pERK). Here, we chose the fear-related infochemical cadaverine (Hussain et al., 2013), a death-associated odour, as an olfactory cue. Cadavarine has been shown to increase the level of pERK in OSNs and induce a robust olfaction-mediated avoidance behaviour in zebrafish (Dieris et al., 2017; Hussain et al., 2013). Consistent with GCaMP data, EE2 showed reduced basal and cadavarine-evoked pERK levels in 5 dpf ERE:nfsBmCherry larvae, whereas EROB cell ablation or ICI induced the opposite effect: the pERK levels in the OB with and without cadavarine were higher than those in controls (Fig. 7A,B). These data support the idea that estrogen inhibits odour-mediated neuronal activation through EROB cells (Fig. 7A,B). Finally, we examined the impact of altering estrogen signalling by EE2 or ICI on olfaction-mediated behaviour. Using a video tracking system, we found no location preference for zebrafish larvae in either control, EE2 or ICI groups during the acclimation period, indicating no differences in swimming behaviour capability among the experimental groups (Fig. 7C, left column; Fig. 7D). After the administration of cadaverine, avoidance behaviour was seen in the control group only (Fig. 7C, right column; Fig. 2E). Conversely, neither EE2- or ICI-exposed larvae showed a clear location preference before or after cadavarine administration (Fig. 7C-E). In this system, we could not determine whether EROB cell ablation also alters olfactory-mediated avoidance response because MTZ treatment alone abrogated a cadavarine-mediated avoidance behaviour. These data suggest that, where the level of local neuronal activity in the OB is either too low (EE2 exposure) or too high (ICI exposure), there was a subsequent suboptimal response to cadavarine, affecting the sensing of an alarm odour and the normal expression of associated fear-related behaviour.

## DISCUSSION

### OB as a primary target of the estrogen signalling pathway in the zebrafish embryonic brain

Here, we showed that the OB in the forebrain is a primary target of the estrogen signalling pathway in the zebrafish embryo. At the developmental stages that we used for this study, the endogenous estrogen activity in estrogen-responsive tissues is not detectable in our ERE:TG models (Green et al., 2016; Lee et al., 2012a) (Fig. S5B). This is likely because of a combination of the very low levels of endogenous estrogens in embryo/larval stages and detection sensitivity limits of the TG lines. However, with exogenous EE2 exposure, estrogen-responsive cells are seen in several tissues (e.g. the liver, heart and somite muscles) (Green et al., 2016; Lee et al., 2012a; Moreman et al., 2017) (Fig. S5A) and a small number of glial cells in the OB (EROB cells; this study). EROB cells are one of the earliest cells that occur in EE2-exposed ERE:TG embryos, which are detectable from shortly after the regional specification of the embryonic brain (i.e. 27 hpf) up to 10 dpf (Fig. 1). We confirmed that exposure conditions used in this study do not globally affect embryo development, overall brain development (Fig. S5A,B) or the expression domains of two different early neural cell markers (Elavl3 and Sox2) (Fig. S6), suggesting roles of estrogens during embryonic brain development are likely specific to the OB. Interestingly, the ontogenic profiles of EROB cells coincide with the time window for olfactory glomerular development in the zebrafish embryo/larvae (Fig. 1J) (Braubach et al., 2013; Li et al., 2005; Miyasaka et al., 2013). In contrast to the OB-specific estrogen responsiveness seen here, the expression domains of ERs are not specific to the OB but rather occur more widely across the CNS in zebrafish embryos from 24 hpf onwards (Tingaud-Sequeira et al., 2004). At 48 hpf, expression of ESR1 (zebrafish homolog of ERα) is greatly reduced in the brain whereas ESR2a (zebrafish homolog of ERβ) is more specifically expressed in the diencephalon and ESR2b (zebrafish homolog of ERβ) in the telencephalon, preoptic area and hypothalamus (Mouriec et al., 2009). Currently, which of the ER isotype(s) is expressed in the OB of zebrafish embryos is not known. Furthermore, wondering why we detect such a localised estrogen activity in our ERE-TG lines given the widespread distribution of ERs from 24 hpf, we think the OB-specific estrogen activity may be attributed to the brain-region-specific expression of *cyp19a1b* (brain-specific aromatase). We show that EROB cells express *cyp19a1b* (Fig. 2Bi,Bii). It is likely that *cyp19a1b* possesses a brain-region-specific enhancer in conjunction with the ERE in its transcriptional regulatory regions (Tong and Chung, 2003) and thereby could locally produce estrogens through an autocrine mechanism. The estrogen signalling cascade in EROB cells could therefore be amplified by a positive feedback via their own aromatase activity. It is noteworthy that, unlike the *cyp19a1b:GFP* transgene, which includes 3.4 kb of the endogenous *cyp19a1b* promoter region (Tong et al., 2009), the *ERE:Gal4ff* transgene in our ERE-TG models contains three tandem-repeated short EREs combined with a TATA minimal promotor only. Thus, the ERE-TG models can detect ER-mediated transcriptional activities widely throughout the embryo without being affected by tissue-specific enhancers or suppressors (Fig. S3A) (Lee et al., 2012a). To date, the use of various estrogen biosensor zebrafish embryos exposed to estrogens has identified several other estrogen-responsive brain cells, located in the olfactory epithelia, telencephalon, preoptic area and hypothalamus in embryo-larvae (i.e. 4-5 dpf) (Brion et al., 2012; Gorelick and Halpern, 2011). However, the roles of these cells in embryonic brain development are largely unknown. In EE2-exposed ERE:TG embryos at 4 dpf we found that estrogen activity predominantly occurs in EROB cells, as we show in whole brain *z*-projection images (Fig. S5B). This provides strong evidence that the EROB cells are a major focus for estrogen activity at the earliest stage of embryonic brain development, which is likely to be enhanced locally through the aromatase-mediated positive feedback mechanism. EROB cell ablation experiments using MTZ supported a crucial role of EROB cells in OB development and function, albeit this effect via EROB cell ablation was less acute than that induced by ICI exposure (Figs 4B and 6F,G). This may, at least in part, be because we were unable to achieve complete EROB cell ablation using MTZ (Fig. S3A,B). More complete cell ablation (e.g. via single cell laser ablation) of all EROB cells would clarify whether the effects of estrogens on OB development and excitability are solely dependent on the EROB cells or whether other estrogen-responsive brain cells might also have some influences on these OB-specific estrogen-mediated functions.

Interestingly, previous descriptive analyses using rat embryonic brain sections demonstrated that the termini of embryonic radial glia cells (RGCs) intermingle with OSN axons to form the foundation of OB glomeruli, protoglomeruli, during early embryonic brain development (Bailey et al., 1999; Ramón-Cueto and Valverde, 1995; Valverde et al., 1992). These observations support a conserved developmental mechanism for the estrogens/EROB cell cascade between fish and mammals. In fact, EROB cells may be a type of such embryonic RGCs given that they express a brain-specific aromatase (*cyp19a1b*) that is known to be expressed specifically in a subpopulation of RGCs in zebrafish (Brion et al., 2012; Menuet et al., 2005). We found that suppressing estrogen signalling by ER antagonist or EROB cell ablation caused developmental abnormalities in key components of the OB glomeruli, including the EROB cells, OSNs and inhibitory synapses (Figs 3-5): in ICI-treated embryos, EROB cell projection networks are not established (Fig. 3). Without the EROB projection networks, the topology and the size of OB glomeruli are markedly altered (Fig. 4). Such defects in organisation of OB glomeruli can consequently impair the establishment of inhibitory synapses in OB glomeruli, as shown in ICI-treated embryos (Fig. 5). It is, however, possible that the estrogens/EROB cascade may also impact on development of other components of the OB glomeruli, such as mitral cells, interneurons and excitatory synapses. Addressing such possibilities would be an important next step to further define the roles of estrogens/EROB cells in the OB development.

### Estrogens and EROB cells establish the responsiveness of the embryonic olfactory system

We found that the OB-specific roles of EE2/EROB cells in olfactory glomerular development contribute also to neurophysiological processes: a developmental exposure of zebrafish embryos with EE2 inhibits the intrinsic excitability predominantly in the OB, whereas ICI activates the intrinsic excitability exclusively in the OB (Fig. 6). The intrinsic neuronal activities in other brain regions are not affected by EE2 or ICI (Fig. 6). It is important to note that previous work has shown that, in zebrafish embryos, elavl3 promoter induces a fluorescent reporter protein predominantly in mitral cells, which is an excitatory output neuron in the OB (Li et al., 2005). We therefore think that GCaMP signals in the OB that we observe here originate most likely from the excitatory input in the OB, not the inhibitory input from interneurons. We further show that EE2 impairs olfaction-mediated neuronal activation in the OB, whereas EROB cell ablation cancelled the effect of EE2. Together, these data suggest that the estrogens/EROB cascade modulates local excitability in the OB during embryonic brain development.

Olfaction is essential for survival, for behaviours such as feeding, mating, social interaction and assessing dangers. Zebrafish embryos hatch after 48 hpf and start feeding at 4-5 dpf and odour-induced neuronal activation in the OB can be detected from as early as 2.5-3 dpf (Li et al., 2005). Olfaction-mediated behavioural responses are observed from 4-5 dpf (Lindsay and Vogt, 2004). We found that EE2-treated animals show impaired olfaction-mediated avoidance behaviour (Fig. 7), which is consistent with its effect on both intrinsic and odour-evoked excitability (Figs 6 and 7). Interestingly, however, ICI-treated animals also exhibited a loss of olfaction-mediated avoidance behaviour (Fig. 7). This may be due to the elevated basal neuronal activity in ICI-treated embryo/larvae as seen in the GCaMP and pERK assays (Figs 6 and 7). The failure in the odour-evoked behaviour response in the EE2- or ICI-treated larvae may imply that the signal/noise ratio with and without an odour substance may need to be above a certain threshold to drive the appropriate behaviour response (Fig. 7; Fig. S7).

Based on these results, we propose that the estrogens/EROB cascade plays a key instructive role in the development of olfactory glomeruli in the OB: estrogens primarily activate the growth of the EROB cell projection networks in the OB. EROB cells in turn act as a scaffold that facilitates the establishment of olfactory glomeruli including the local inhibitory circuits. Consequently, the estrogens/EROB cell cascade controls the intrinsic spontaneous and odour-evoked excitability in the OB, influencing odour sensing and associated behaviour.

### Estrogens/EROB cascade – a potential site of action for EDCs or a possible link to neurodevelopmental disorders

A wealth of data exist on the effect of EDC exposure on olfaction-mediated responses, including on odour-induced neurogenesis, excitability and olfaction-linked cognition/memory and behaviour in mammals including humans (Brus et al., 2016; Cherian et al., 2014; Doty et al., 2008; Kollndorfer et al., 2016; Veyrac and Bakker, 2011). These studies, however, have primarily focused on adult stages of humans and rodents, with very little attention given to the role of estrogens in the development of the olfactory sensory system. Our data highlights the possibility that the estrogens/EROB cell cascade in early embryonic stages could be an important site of action for EDCs in the environment to interfere with the endogenous functions of estrogens. If so, exposure to environmental estrogens during embryonic brain development could cause adverse effects on the olfactory sensory system and impact on fitness and social/sexual behaviour in later life. In fish, chemical communication through olfaction also plays a crucial role in shaping ecological interactions, allowing animals to locate food sources, predators, habitats or mates (Wyatt, 2003). Given that the developing embryo is generally the most susceptible life stage to environmental toxicants, this possibility warrants further investigation.

Furthermore, alterations in the estrogens/EROB cascade during development might link with certain neurodevelopmental disorders (i.e. ADHD and autism) (Crider and Pillai, 2017; Mustieles et al., 2015). Notably, the clinical symptoms for these conditions are often associated with olfactory impairment (anosmia) and/or altered olfactory sensitivity (Endevelt-Shapira et al., 2018; Sundermann et al., 2008; Valdes-Socin et al., 2014). Interestingly, estrogenic compounds have been shown to selectively rescue the behaviour phenotype observed in the *contactin associated protein-like 2* (*cntnap2*) mutant zebrafish embryo (double mutant of *cntnap2a* and *cntnap2b*, termed *cntnap2ab* mutants), an autism-related gene mutant line carrying GABAergic neuron deficits (Hoffman et al., 2016). Thus, examining the estrogens/EROB cell cascade in *cntnap2ab* mutants could give a new insight into the molecular/cellular mechanism underlying the pathogenesis of neurodevelopmental disorders.

In summary, our work identifies a fundamental role of estrogens in development of the olfactory sensory system in the embryonic brain, which could result in a long-lasting influence on neuronal circuitries responsible for olfaction and could impact on behaviour and fitness in later life. Further research is required to determine whether this estrogens/EROB cell cascade is conserved across vertebrate species. The identification of specific target genes involved in the estrogens/EROB cell cascade would be the next step to elucidate a precise mechanism of action of estrogens in EROB cells.

## MATERIALS AND METHODS

### Fish husbandry and TG experiments

All experimental procedures conducted in this research with zebrafish were in accordance with U.K. Home Office regulations for the use of animals in scientific procedures and followed local ethical review guidelines ensuring their humane treatment. See Table S1 for TG zebrafish lines used in this study and supplementary Materials and Methods for further details of the experimental zebrafish lines.

### Chemical exposure

#### Developmental exposure to EE2 or an estrogen receptor antagonist (ICI)

To examine the roles of the estrogen signalling pathway in brain development, zebrafish embryos collected from the selected transgenic zebrafish lines, depending on the requirement for the different experiments, were exposed to 100 ng/l ethinylestradiol (EE2, Sigma-Aldrich) or 10 µM ICI 182,780 (ICI, Sigma-Aldrich) (aqueous exposure in system water) from 1 to 96 hpf. For the dose response experiments to these compounds (Fig. 6C), zebrafish embryos were exposed to 25, 50 or 100 ng/l EE2 or to 2.5, 5 or 10 µM ICI. For pERK assay and the olfaction behaviour assay, the exposure period to these chemicals was extended to 5 days (up to 120 hpf).

#### Transient exposure to EE2 or ICI

For testing the acute effect of exposure to estrogen (EE2) or the estrogen receptor antagonist (ICI) on neuronal activity (Fig. S5), *elavl3:GCaMP6s* embryos were cultured in aquarium system water without chemical exposure up to 96 hpf. At 96 hpf, before imaging with light sheet microscopy (LSM), embryos were exposed to 100 ng/l EE2 or 10 µM ICI in 1× E3 media for 20 min at room temperature. Following exposure, GCaMP6s signals in each embryo were examined by LSM, as described below.

#### Chemical exposure conditions for chemical/genetic ablation of EROB cells in ERE:mCherry line

To induce selective ablation of EROB cells, we employed nitroreductase-mediated chemical/genetic cell ablation (Curado et al., 2008). In the ERE:mCherry line, Tg(*ERE:Gal4ff; UAS*:*nfsBmCherry*), intracellular nitroreductase synthesis is under control of estrogen-mediated ERE-activation. Selective ablation of EROB cells was achieved by applying EE2 together with a prodrug MTZ (Sigma-Aldrich) to induce cytotoxic metabolites only in estrogen-responsive cells. ERE:nfsBmCherry embryos were exposed to either aquarium system water (control), 100 ng/l EE2, 7.5 mM MTZ or 100 ng/l EE2 plus 7.5 mM MTZ (EE2+MTZ) from 1 to 33 hpf. The timing (from 1 hpf to 33 hpf) for EE2+MTZ treatment is the time-window when EROB cells have just started developing (Fig. 1E) and estrogen-responsive cells in other tissues such as the liver and the muscle are still very limited in their development. We designed this exposure to minimise the possible indirect effects on OB development/function and/or global development caused by ablation of other cells expressing the nitroreductase enzyme under ERE promoter activity. We confirmed that this exposure was able to achieve effective ablation of EROB cells in the absence of developmental abnormalities (∼70% reduction of EROB cells at 4 dpf; Fig. S3A,B). Following the chemical exposures, embryos at 33 hpf were washed once and cultured in aquarium water until 4 dpf and subsequently used for experiments.

#### Mosaic expression of DsRed in a subset of EROB cells

*pT2A UAS:DsRed-Ex* plasmid DNA (Miyasaka et al., 2014) was injected into one-cell-stage ERE:GFP embryos. See further details in supplementary Materials and Methods.

### EROB cell projection analyses

To examine the effect of estrogens on cellular projections of the EROB cells, ERE:GFP embryos were treated with 100 ng/l EE2 from 1 hpf to 72 hpf to induce GFP in EROB cells. Subsequently, EE2 was washed off from the media and the embryos underwent either non-chemical incubation from 72 hpf to 120 hpf [control (EE2 washed off)] or exposure to 10 µM ICI to inhibit estrogen activity from 72 hpf to 120 hpf [ICI (from 72 to 120 hpf)]. At 120 hpf, both groups of embryos were mounted in 0.7% low melting point agarose in a 35 mm diameter glass-bottom dish (MatTek) with the embryo angled at ∼40° dorsal surface facing downwards. Confocal images of EROB cells were obtained using Zeiss 880 Airyscan panel acquisition with 40× objective. Images were Airy-processed and stitched using Zeiss Zen Black software and further processed using Fiji with set parameters for brightness/contrast adjustment and background subtraction. These images were then presented as maximum intensity projection images. To quantify the effect of ICI on the EROB cell projection networks, mean grey values of GFP signals within the margins of the EROB cells (as marked with a green square in Fig. 3Ai,Bi) were obtained using the surface plot function in Fiji. Mean GFP signals within the EROB cell margin were also plotted along the medial-lateral *x*-axis, normalising measured *x*-co-ordinates by setting the position of the midline to 0 and the lateral ends to 1 (right) and −1 (left) (*n*=6 for each). The average of grey values and sum of grey values (% of total grey value in each embryo) in left lateral end [*x*=−1 to −0.7] and right lateral end [*x*=0.7 to 1] of the above plots were analysed to examine the effect of ICI on EROB cell projection networks.

#### Statistics

Statistics were performed with R (version 3.6.1) (R Core Team, 2019). A Shapiro-Wilk test identified that the data for the average of grey values in both left and right margins were not normally distributed and applying Levene's test showed heterogeneity of variances. Thus, a non-parametric Mann–Whitney *U*-test was performed. The same statistical analysis was also applied for the percentage of total grey value in both margins. The EROB cell count showed normal distribution and homogeneity of variances, thus a *t*-test was performed to explore the effect of ICI on the number of EROB cells.

### Whole-mount immunohistochemistry

All whole-mount immunohistochemistry was conducted with 4% paraformaldehyde (PFA)-fixed 4 dpf ERE:GFP pr ERE:mCherry embryos with or without chemical exposures as described above. See further details in supplementary Materials and Methods.

### Immunohistochemical analysis for gephyrin-expressing inhibitory synapses

Chemical exposure conditions for ERE:GFP embryos were as described above. At 4 dpf, exposed embryos were anaesthetised with 0.03% NM222 and then embedded in NEG-50 solution (Thermo Fisher Scientific) without PFA fixation, flash frozen in liquid nitrogen and stored at −80°C. Embedded frozen samples were transverse-sectioned in consecutive 25 µm thickness sections using a CM1950 cryostat (Leica). Sections were transferred to SuperFrost Ultra Plus Gold Adhesion Slides (Thermo Fisher Scientific) and post-fixed with 4% PFA/PBS for 15 min at room temperature. Fixed sections were gently rinsed twice with 1× PBS and treated with pepsin reagent (Sigma-Aldrich, R2283) for 5 min at 37°C, followed by carefully rinsing twice with 1× PBS. Subsequently, the samples were permeabilised with 0.1% Triton/PBS for 3 min at room temperature and then rinsed twice with 1× PBS and blocked with 2% bovine serum albumin/0.2% milk in PBS for 1 h at room temperature. Blocked sections were subsequently stained with mouse anti-gephyrin antibody (1:500 in the same blocking solution, Synaptic Systems, #147011) and rabbit anti-GFP antibody (1:500 in blocking solution, AMS Biotechnology, TP401) at 4°C overnight. The samples were then washed three times with 1× PBS and incubated with Alexa 594-conjugated anti-mouse IgG antibody (1:500 in blocking buffer, Thermo Fisher Scientific, A11012) and Alexa 488-conjugated anti-rabbit IgG antibody (1:500 in blocking buffer, Thermo Fisher Scientific, A11034) for 1 h at room temperature. The stained samples were then washed three times with 1× PBS and incubated with Hoechst 33342 (1:25,000 in 1× PBS, Thermo Fisher Scientific) for 20 min. Finally, the samples were washed twice with 1× PBS and mounted with ProLong Gold antifade reagent (Thermo Fisher Scientific). Stained sections were imaged using Airy scan Zeiss LSM880 with a 20× or 40× objective lens. The region covering the whole OB was captured from three or four consecutive (25 µm thickness) sections per individual sample. Images were Airy-processed and/or stitched (for panel acquisition images) using Zeiss Zen Black software and further processed with Fiji with a set parameters for brightness/contrast adjustment and background subtraction. Gephyrin puncta were quantified by applying a threshold (IJ_IsoData method) and watershed algorithm to obtain binary images of gephyrin+puncta and selecting only those ranging in the sizes 0.1-0.4, 0.41-0.99 and 1-10 µm^2^ using the analyse particles function in Fiji. The outlined drawings of the size-selected puncta were merged with the nuclei staining image of the section to verify the positions of the puncta in the OB regions. Positions of the OB in the brain sections were confirmed in accordance with the atlas of early zebrafish brain development (Mueller and Wullimann, 2015). The number of puncta in the OB regions was counted manually using multipoint function in Fiji. Gephyrin puncta numbers in each puncta size-range group were summed and displayed as percentage of total number in control (*n*=4 each).

#### Statistics for gephyrin puncta analysis

Statistics were performed with R (version 3.6.1) (R Core Team, 2019). Normality of the data was confirmed with the Shapiro test. Levene's test showed homogeneity of variances and a linear model was built. A one-way ANOVA, in conjunction with Tukey's post-hoc test, was performed for pair-wise comparisons of the treatments, using the ‘multcomp’ package in R.

### GCaMP6s imaging

#### Experimental settings for GCaMP6s imaging using light sheet microscopy

The detailed procedures for sample preparation for GCaMP6s imaging and for imaging acquisition using a custom-built LSM are described in Winter et al. (2021, 2017). A full brain volume of GCaMP6s images for zebrafish embryo larvae was captured in around 1.8 s (10 horizontal plane optical *z*-slices in 24 μm steps). These were taken repeatedly for ∼6 min from which the ROI-specific neuronal activity data were extracted. We included 41 anatomically registered brain ROIs, which encompassed all the major brain structures of relevance. ‘Peak value’ of GCaMP6s signals in an ROI were compared statistically between control and experimental groups (*n*=8 for each). ‘Peak value’ represents the average of the peaks of GCaMP6s signals within that ROI over the experimental period and are considered to be the most revealing parameter for showing changes in the intrinsic neuronal activity within each brain region. To minimise experimental subject variability in GCaMP6s fluorescence, *elavl3:GCaMP6s* embryos were pre-screened for a similar basal GCaMP expression level in the brain before LSM imaging. All experimental treatments were conducted within one batch of embryos, and repeated on two separate occasions, to account for possible batch-to-batch variations and all imaging conditions were kept identical throughout the study.

#### Statistics for LSM GCaMP data

Statistics were performed with R (version 3.2.3-4). The LSM data were analysed using a generalised linear model with ‘Treatment’, ‘Region of interest (ROI)’ and the interaction of both as fixed effects. A Shapiro-Wilk test showed that the data were not normally distributed (*P<10^−16^) and the box cox normality plot suggested a logarithmic correlation. Therefore, a gamma distribution with a logarithmic link was used to fit the continuous data. The likelihood ratio test in conjunction with Tukey's post-hoc test was then performed using the estimated marginal means ‘emmeans’ package in R.

#### GCaMP imaging using Zeiss Airyscan

To analyse neuronal activity under the condition of EROB cell ablation, heterozygous embryos of Tg(*ERE:Gal4ff; UAS*:*nfsBmCherry*)×Tg(*elavl3:GCaMP6s*) were produced from a pair-cross of homozygous parent of each TG fish. Chemical exposure conditions for EROB cell ablation and for inhibiting ER activation by ICI (10 µM) are described above. An efficient ablation of EROB cells were confirmed, as shown in Fig. S3C. We emphasise that this system had a reduced (50%) sensitivity for GCaMP6s imaging, compared with that of homozygous Tg(*elavl3:GCaMP6s*), due to the heterozygosity of the transgenes. To minimise the variation, all datasets were acquired on the same date using the same batch of embryos (*n*=7 in each experimental group). The same experiments were repeated twice with comparable results. At 96 hpf, exposed embryos were washed once with 1× E3. Each embryo was treated with 4 mM anti-nicotinic neuromuscular blocker tubocurarine (4 mM; Sigma-Aldrich) until muscle tone was lost. The immobilised embryo was then quickly mounted in 0.7% low melting point agarose in a 35 mm diameter glass-bottom dish (MatTek) with the embryo angled at ∼40° with the dorsal surface facing downwards. Live imaging of the *elavl3*:GCaMP6s signal was carried out using Zeiss 880 in fast acquisition mode with Airyscan, which achieves nine consecutive optical *z*-section images extending through the entire OB (scan depth 63 µm, 7 µm step each) in 2.2 s, allowing active neurons labelled with *elavl3*:GCaMP6s throughout glomerular layer to be detected. This approach allowed individual cell-level functional imaging to be undertaken at a similar temporal resolution to that obtained using LSM, but across a lower *z*-depth. This was nonetheless appropriate for imaging the OB region, rather than the whole brain. Image acquisition was repeated for 50 cycles, generating 50×9 optical *z*-section images. *Z*-projection images of EROB cells (ERE:mCherry) were also obtained from the same experimental embryos to confirm nitroreductase-mediated EROB cell ablation.

#### Data processing of Airyscan GCaMP6s images and statistics

Raw data for *elavl3*:GCaMP6s images from each experimental embryo were processed in Fiji to obtain a time series of average intensity *z*-projection images (Ave_projections). A reference fluorescence intensity level corresponding with the basal activity in each fish was obtained by randomly selecting three areas at the edge of the OB in which the signals from Ave_projections over ∼2 min remained consistently lower than the signals in the OB glomeruli. Baseline fluorescence (F) of each fish was obtained by averaging the raw signals from the three chosen reference areas. No significant change in F was observed in all treatment conditions (Fig. 6E). ROIs for the medio-dorsal glomeruli and the entire OB were manually selected using the polygon selection tool. Functional signals in these ROIs were extracted from the time series of mean grey values in Ave_projections and shown as dF/F(%) [percentage of the change in the fluorescence (dF) normalised to the baseline fluorescence (F) for each fish]. No significant time-window deviation in dF/F(%) was seen in all treatment conditions (Fig. 6H). Data were analysed with one-way ANOVA in conjunction with Tukey's post-hoc test for multiple comparisons, using GraphPad Prism version 9.2.0.

### pERK assay

Chemical exposure to ERE:mCherry embryos was performed as described above. At 5 dpf, the samples were stimulated with or without 100 μM cadavarine (D22606, Sigma-Aldrich) for 5 min, then fixed and embedded for cryosectioning. Sections that cover the OB area of the samples were stained with anti-phospho p44/42 MAP Kinase (ERK1/2) rabbit polyclonal antibody (4370, Cell Signaling Technology) to analyse basal or odour-evoked neuronal activation. For further details, see supplementary Materials and Methods.

### Olfactory behaviour assay

Chemical exposure to ERE:mCherry embryo was performed as described above. At 5 dpf, EE2- or ICI-exposed embryos were washed and cultured in a glass dish containing 75 ml of aquarium system water at a density of 30 larvae/75 ml water and were then fed until the day of the olfaction behaviour assay at 9-11 dpf. One-third of the volume of incubation water was changed daily until the time of the behaviour assessment. Larval movement was measured using automated videotracking (ViewPoint; Readman et al., 2013; Winter et al., 2008) in an experimental arena (indicated in Fig. 7C, control-cadavarine image, top-right). Each larva was assessed before testing for olfactory responsiveness to ensure an appropriate level of spontaneous swimming behaviour. This was achieved by placing each larva into the experimental arena, which contained 45 ml of fresh aquarium water, and recording its swimming behaviour for a 5 min acclimation period at 25 frames per second (fps). Individuals entering more than seven areas without a location bias or more than six areas with a location bias to the centre of the chamber (the location areas in the experimental chamber are indicated with lines in Fig. 7C) were considered appropriate for the olfactory test. Subsequently, 200 µl of 1 mM cadavarine (D22606, Sigma-Aldrich) was introduced gently through a silicon capillary tube into one side of the experimental arena, while the same amount of water was simultaneously applied to the opposite side of the chamber. This produced a cross-arena biased gradient of cadavarine with minimum disturbance of the animal. During method development, it was established that the gradient of a chemical was maintained throughout the 5 min of recording based on the diffusion rate of Phenol Red (data not shown). The swimming behaviour of the larva after cadavarine administration was recorded for a further 5 min and the data analysed for the time spent within the cadaverine-treated (red highlighted) or -untreated areas (blue highlighted) using Viewpoint software. The cadavarine-mediated avoidance response was defined as the average duration spent (residence time) in the red (cadavarine high) versus blue (cadavarine low) areas (Fig. 7E).

#### Statistics for behaviour data

Statistics were performed using R (version 3.2.3-4). The paired experimental design and thus dependency of the test parameters on the individuality of each zebrafish larva required a mixed effect model analysis approach using the ‘individual’ as a random effect. The distribution of the data was best described with a normal distribution, leading to the choice of a linear mixed effect model, using the ‘lme4’ package in R. Fixed effects of this model were Treatment and Area as well as their two-way interaction. The date of the experiment and the developmental stage (9-11 days) had no significant effect on the model. For pair-wise comparisons within the treatment groups, Tukey's test was performed using the ‘emmeans’ package in R.

## Supplementary Material

Supplementary information

Reviewer comments
