# Supplementary material for: Estrogens regulate early embryonic development of the olfactory sensory system via estrogen-responsive glia
Source: Development. 2022 Jan 13;149(1):dev199860. doi: 10.1242/dev.199860 (PMC8881738; doi:10.1242/dev.199860)
Supplement: Supplementary information [file develop-149-199860-s1.pdf]

Table S1. List of TG zebrafish lines employed

TG lines	TG line names in the shorter form	Experiments for the use	Stage used for experiments
Tg(*elavl3*(*huC*)*:GCaMP6s*)	elavl3:GCaMP6s	4D functional imaging of neuronal activity using light sheet microscopy	4 days post fertilization (4 dpf)
Tg(*ERE:Gal4ff; UAS:GFP*)	ERE:GFP	Characterisation and ontogenic profiling of EROB cells	1-21 dpf
Tg(*ERE:Gal4ff; UAS nfsBmCherry*)	ERE:mCherry	Chemical/genetic ablation of EROB cells	4 dpf or 5 dpf
Tg(*ERE:Gal4ff; UAS nfsBmCherry*)	ERE:mCherry	Olfaction-mediated behaviour assay	9-11 dpf
Tg(*ERE:Gal4ff; UAS nfsBmCherry; elavl3:GCaMP6s*)	ERE:mCherry x elavl3:GCaMP6s	Chemical/Genetic ablation of EROB cells and GCaMP imaging by confocal microscopy	4 dpf
Tg(*ERE:Gal4ff;UAS nfsBmCherry;cyp19a1b:GFP*)	ERE:mCherry x cyp19:GFP	Characterisation of EROB cells for co-localisation of a brainspecific aromatase B	4 dpf

Development • Supplementary information

Supplementary Materials and Methods

Experimental zebrafish lines

The elavl3(*huC*):GCaMP6s transgenic zebrafish line, Tg(*elavl3:GCaMP6s*), used in this study has been described previously in (Winter et al., 2017), and was originally supplied by Misha B. Ahrens (Janelia Research Campus, Howard Hughes Medical Institute, Ashburn, Virginia, USA). The ERE:GFP line, Tg(*ERE:Gal4ff; UAS:GFP*), carrying two transgenes, *ERE:Gal4ff* and *UAS:GFP*, in a skin pigment free Casper background was developed at the University of Exeter (Green et al., 2016). ERE:mCherry line, Tg(*ERE:Gal4ff; UAS*:*nfsBmCherry*), was created for this study encoding an ERE-derived Gal4ff (*ERE:Gal4ff*) and a UAS-derived mCherry-nitroreductase fusion protein (*UAS:nfsBmCherry*). This was developed from our original ERE:GFP line by replacing *UAS:GFP* with *UAS:nfsBmCherry*. Briefly, homozygous Tg(*ERE:Gal4ff; UAS:GFP*) zebrafish and a TG zebrafish carrying *fms:Gal4* and *UAS:nfsBmCherry* (originally supplied by Stephen A. Renshaw)(Gray et al., 2011) were pair-crossed to obtain offspring heterozygous for these four transgenes. The heterozygous offspring were raised to sexual maturity and in-crossed. From this in-crossed generation, embryos were collected and exposed to 50 ng/L 17α-ethinyloestradiol (EE2)(Sigma-Aldrich) from 0 to 3 days post fertilization (dpf) and screened for estrogen-dependent mCherry expression in estrogen responding tissues (Green et al., 2016; Lee et al., 2012) with an absence of either GFP expression in these tissues (*UAS:GFP* null) or mCherry expression in macrophages (*fms:Gal4* null). These screened larvae were raised to sexual maturity and further in-crossed and following this F2 embryos were again screened for estrogen dependent mCherry expression and absence of other TG expression, as described above. Finally, homozygous progeny of Tg(*ERE:Gal4ff; UAS*:*nfsBmCherry*) was confirmed by pair-crossing with wild-type (WIK strain) adults and assessing the ratio of fluorescent/non-fluorescent offspring, subsequently establishing Tg(*ERE:Gal4ff; UAS*:*nfsBmCherry*), the ERE:mCherry line.

Development • Supplementary information

Tg(*ERE:Gal4ff;UAS:nfsBmCherry;cyp19a1b:GFP*), the ERE:mCherry x cyp19:GFP line, was generated by natural-crossing of ERE:mCherry and cyp19a1b:GFP zebrafish (Brion et al., 2012)(the latter generously provided by Olivier Kah). The homozygous for three transgenes (*ERE:Gal4ff, UAS:nfsBmCherry and cyp19a1b:GFP*) were screened using a similar procedure to that described for the homozygous screening for ERE:mCherry.

Mosaic expression of DsRed in a subset of EROB cells.

To map the morphology of an individual EROB cell, *pT2A UAS:DsRed-Ex* plasmid DNA (Miyasaka et al., 2014) (25 ng/μl) and *Tol2 transposase* mRNA (25 ng/μl) were co-injected into one cell stage ERE:GFP embryos and subsequently exposed to EE2 (100 ng/L) from 1 to 96 hpf. This rendered a mosaic expression of DsRed in a subset of oestrogen responding cells which was derived from oestrogen-dependent ERE:Gal4ff activation in these cells. The morphology of DsRed expressing EROB cells at 4 dpf was examined using a Zeiss LSM510 confocal microscope.

Immunohistochemistry

Whole mount GFAP, Acetylated tubulin, SV2, Hemocyanin and mCherry staining:

The zebrafish embryo-larvae chemical exposure conditions are described in the “Methods” of the main manuscript. At 96 hpf, exposed embryo-larvae were washed, anaesthetised with 0.03% NM222 and fixed in 4% paraformaldehyde (PFA) in 1 x PBS at 4°C overnight. Fixed samples were then washed once with 0.5 % Triton in 1 x PBS (PBTr) and transferred to 50% methanol / 50% PBTr for 5 minutes, then to 100% methanol for 5 minutes, followed by placing them into fresh 100% methanol at -20°C (for between overnight - 2 weeks). The samples were then rehydrated with 50% methanol / 50% PBTr for 5 minutes and subsequently with PBTr for 5 minutes. Embryos were then treated with 3 x proteinase K solution (Sigma-Aldrich) (final 30 μg/ml) for 30 minutes and then washed three times with PBTr and blocked with 5% FBS, 2.5% BSA, 1% DMSO in PBTr at 4°C overnight. Blocked embryos were incubated with the following primary antibodies at 4°C overnight: mouse anti-GFAP antibody (1:5000 in blocking solution, ZIRC, zrd-1); rabbit anti-mCherry antibody (1:300 in blocking, abcam, ab167453); mouse anti-acetylated tubulin antibody (1:300 in blocking, Sigma-Aldrich, T7451); and mouse anti-SV2- antibody (1:200 in blocking, DSHB, SV2-s) and rabbit anti-hemocyanin (KLH) antibody (1:200 in blocking, Sigma-Aldrich, H0892) or mouse anti-mCherry antibody (1:200 in blocking, St. John’s Laboratory, STJ34373). The incubation period for primary antibodies against SV2 and KLH was extended to a week (at 4°C) in order to

Development • Supplementary information

increase staining efficiency. The embryos were washed three times for 30 minutes with PBTr and incubated with Alexa 594 conjugated anti-mouse IgG antibody (1:300 in blocking, ThermoFisher Scientific, A11012) and Alexa 488 conjugated anti-rabbit igG antibody (1 : 300 in blocking, ThermoFisher Scientific, A11034), or with Alexa 488 conjugated anti-mouse IgG antibody (1:300 in blocking, ThermoFisher Scientific, A11001) and Alexa 594 conjugated anti-rabbit IgG antibody (1:300 in blocking buffer, ThermoFisher Scientific, R37121) at 4°C overnight. The embryos were washed three times for 30 minutes with PBTr, and once with 1 x PBS for 10 minutes and then incubated with Hochest 33342 (1:3000 in 1 x PBS, ThermoFisher Scientific) for 30 minutes at room temperature to stain the nuclei. The stained embryos were washed twice for 10 minutes with 1 x PBS at room temperature and kept in 50% Glycerol in 1 x PBS at 4°C until imaging.

Whole mount HuC (Elavl3), SOX2 and GFP staining:

Chemical exposure conditions for ERE:GFP embryos were performed as described in "Methods" of the main manuscript. Fixation, permeabilisation and blocking of the embryos were conducted as described above. The blocked embryos were incubated with mouse anti- HuC antibody (the antibody against human ELAVL3 protein) (1:200 in blocking solution, ThermoFisher Scientific, A21271, 16A11) and rabbit anti-GFP (1:500 in blocking, AMS Biotechonology, TP401), or rabbit anti-SOX2 antibody (1 : 100 in blocking solution, GeneTex, GTX124477) and mouse anti-GFP (1 : 500 in blocking solution, GeneTex, GTX82564) at 4°C overnight. The embryos were then washed and incubated with secondary antibodies and Hoechst, as described above.

Cryosection sample staining for OSNs and EROB cells:

The conditions for chemical exposure and for sample fixation were performed as described in the main manuscript Methods. Fixed embryos were cryo-protected in 30% sucrose in 1 x PBS and embedded in NEG-50 solution (ThermoFisher Scientific). Frontal sections of the forebrain of 4 dpf ERE:mCherry embryo (7 μm thickness) were obtained using CM1950 cryostat (Leica). Sectioned samples were rehydrated with 0.2 % Triton in 1 x PBS (PBTr0.2) and blocked with 0.25 % milk in PBTr0.2 at room temperature for one hour. Blocked samples were then stained with rabbit anti-KLH (1:500 in the same blocking solution, Sigma-Aldrich, H0892) and mouse anti-mCherry antibody (1:500 in blocking, St. John's Laboratory, STJ34373) at 4°C overnight. The samples were washed three times for 15 minutes with PBTr0.2 and incubated with Alexa 594 conjugated anti-mouse IgG antibody (1:500 in blocking, ThermoFisher Scientific, A11012) and Alexa 488 conjugated anti-rabbit IgG antibody (1:500 in blocking, ThermoFisher Scientific, A11034) at room temperature for one hour. The stained samples were then washed three times for 15 minutes

with PBTr0.2 and once with 1 x PBS for 10 minutes and then incubated with Hochest 33342 (1:25,000 in 1 x PBS, ThermoFisher Scientific) for 30 minutes. Finally, the samples were washed twice for 10 minutes with 1 x PBS and mounted with ProLong Gold antifade reagent (ThermoFisher scientific).

Confocal imaging:

Whole mount stained zebrafish embryos and immune-stained cyrosection samples of the zebrafish forebrain were analysed using laser scanning confocal microscopy (Zeiss LSM 510 and Zeiss LSM 810, Zeiss, Germany). For whole mount stained embryos, the samples were mounted in low melting agarose as described above. Confocal z stacks across the entire forebrain or the OBs were obtained using Zeiss LSM510 or Zeiss LSM810 keeping the optimal or at a set z step size.

Co-localisation analysis for EROB cells and GFAP or aromatase B:

Co-localisation of EROB cells and GFAP, or aromatase, were measured using a method described in (Steinfeld et al., 2015). Briefly, Pearson's correlation coefficients in two dimension were calculated between pixel intensities of two distinct channels over a range of image shift (dx) for each volume. Co-localisation of two signals results in a distinct peak at dx = 0. This was compared against control images where the image of one channel was flipped 90° (control flipped image).

3D reconstruction of confocal images and OB glomeruli volume analysis:

The confocal optical z section images were processed to obtain 3D images of the OB glomeruli by 3D Viewer in Fiji. The same data were also processed to quantify the volumes of glomeruli by measuring the areas of ROIs throughout the optical z section images. Briefly, a set threshold (IJ_IsoData method) was applied to raw confocal optical z section images of SV2 stained forebrains, generating binary images of SV2+ glomeruli throughout the z slices. The outlines of five glomeruli (maG, dG, dlG, mdG3 and mdG1-6), which are located in dorsal areas of the OB and interact with EROB cells, were drawn manually using the freehand selection tool. The areas of those outlined glomeruli were measured individually from each z slice and the relative volume of a glomerulus was represented by the sum of the measured areas from the collective z slices. Statistics were performed with R (version 3.2.3-4). Normality of the data was confirmed with the Shapiro test and Levene's test showed homogeneity of variances and a linear model was built. The one-way ANOVA in conjunction with Tukey's post-hoc test was performed for pair-wise comparisons of the treatments, using the 'multcomp' package in R.

pERK assay

pERK immunostaining

The conditions for chemical exposure and for sample fixation were performed as described in the main manuscript Methods. At 5 dpf, ERE:mCherry larvae were exposed with or without 100μM cadavarine (D22606, Sigma-Aldrich) for 5 minutes at 28 °C (5 larvae/1ml of zebrafish egg water/ well of 12 well plate), and then immediately fixed by adding 1ml of 8% PFA in PBS to give final 4% PFA. The larvae in PFA were incubated for 2 hours at 28 °C, and subsequently overnight at 4°C. Fixed embryos were washed three times with tris-buffered saline (TBS) and treated with a sucrose gradient (10 %, 20 %, 30 %) and then stored in 30% sucrose at 4°C for cryoprotection. The samples were embedded in NEG-50 solution (ThermoFisher Scientific) and frozen minimally overnight at -80°C. The forebrain of the mounted larvae were then transversely sectioned at 10 μm thickness consecutively from anterior to posterior using a CM1950 cryostat (Leica). Brain sections were transferred to SuperFrost Plus glass slides, (VWR), air-dried for 1 h and stored at -20°C with silica-gel to remove air moisture.

Prior to staining, the slides were air dried for 1 h and washed with 0.1% Tween 20 in 1x TBS (TBST) for 10 minutes for rehydration. The samples were treated with a gradient of methanol (25, 50, 75 and 100% methanol) in TBST (for 5 min each) and then with 0.3% hydrogen peroxide in 100% methanol for 15 min to block endogenous peroxidase activity and reduce background signals in the Tyramide Signal Amplification (TSA) step (described below). The methanol exposure also removed all detectable ERE-mCherry signal. After washing with TBST, the slides were then washed with heat retrieval solution (Tris-EDTA buffer with 10 mM tris base, 1 mM EDTA, 0.05% Tween 20, pH 9.0) and immersed into a preheated retrieval solution for 10 minutes at room temperature. For antigen retrieval, the slides were then heated to approximately 95°C in heat retrieval solution for 10 minutes. The slides were washed 2 times for 15 minutes in TBS/0.1% Tween 20/0.1% Triton X-100 (TBSTwT) to ensure permeabilisation of the tissues, and then further washed with maleic buffer (150 mM maleic acid/100 mM NaCl/0.05% Tween 20, pH 7.4). Samples were then blocked with maleic blocking buffer (2% blocking reagent (Roche) in maleic buffer) for 1 h at room temperature. The primary antibody, anti-phospho p44/42 MAP Kinase (ERK1/2) rabbit polyclonal antibody (#4370, cell signaling), was then applied at 1:250 in the blocking buffer and the slides incubated overnight at 4°C. The slides were subsequently washed 2 times with TBSTwT, 1 time with maleic buffer and incubated with the maleic blocking buffer for 1h at room temperature for re-blocking. Slides were then incubated with 1:3000 goat anti-rabbit IgG-HRP (Dako) overnight at 4°C. The samples were then washed 3 times with maleic buffer for 30 min and once with TBS for

Development • Supplementary information

15 minutes and then incubated with 1:50 TSA Plus Cyanine 3 (PerkinElmer) in 1x Plus Amplification Diluent for exactly 50 minutes. The enzymatic amplification reaction was terminated by washing 5 time with TBSTwT for total > 4 h at room temperature. The samples were stained with Hochest 33342 (1:25,000 in 1 x TBS, ThermoFisher Scientific) for 20 minutes. Finally, the samples were washed twice with 1 x TBS and mounted with ProLong Gold antifade reagent (ThermoFisher scientific). The stained brain sections were imaged by using inverted light fluorescence microscopy (Zeiss Observer. Z1) with a set light exposure time and filter condition.

Image processing and quantification of pERK+ signals in the OB.

Image processing was performed with Fiji-ImageJ. The same adjustments in brightness and contrast and background subtraction were applied to all images. pERK-postive cells were identified by applying a set threshold (IJ_IsoData method) to obtain binary images of pERK+ signals, selecting only those ranging in the sizes 30-1000 μm^2 using the analyze particles function in Fiji. This size range can include pERK+ signals in a single cell as well as those in olfactory glomeruli in the OB. Total pERK+ signals in the OB were obtained by summing the area sizes of the selected pERK signals from 6-7 sequences of forebrain sections. Positions of the OB in the brain sections were confirmed in accordance with Atlas of early zebrafish brain development (Mueller, 2015). pERK signals were displayed as fold increase over none-treated control. Total sample numbers (N=3-6) were collected from three independent experiments. Fold-increase value was calculated using the average of none-treated control value for each experiment. For alarm substance (AS)-induced pERK assay (Fig. S7A and S7B), 4 dpf ERE:mCherry embryos were exposed with or without EE2 or ICI as described above, and stimulated with 1:2500 AS diluted in zebrafish egg water for 5 minutes, then fixed and used for the following staining procedure as described above. Stained samples were imaged using Zeiss LSM 880 with Airyscan Fast mode. Image processing was performed with Fiji-ImageJ. The same adjustments in brightness and contrast were applied to all images. pERK postive cells were identified as pERK-positive cell bodies with DAPI-positive nuclei in the OB and OE and were counted manually. Total number of pERK positive cells in the forebrain regions were shown (N=2).

Alarm substance (AS) isolation

To isolate alarm substance, we used the method previously described in (Speedie and Gerlai, 2008) with some modifications. Total 5 male wild type zebrafish (AB) were terminated under schedule 1 procedure with overdose of tricaine methane sulfonate (MS222, 400 mg/l) followed by cutting the brain stem with a sharp needle. The excess water on the skin of the fish was

removed with a paper towel. Fifteen shallow cuts on each side of the skin of the donor zebrafish was applied and the cuts were washed in 10 ml Milli Q water on ice. The same procedure was repeated until the cuts from all 5 fish were washed in the same 10ml water. Collected AS was aliquoted in eppen tubes, and the tubes were quickly snap-frozen with liquid N2 and stored at -80°C until use.

Development • Supplementary information

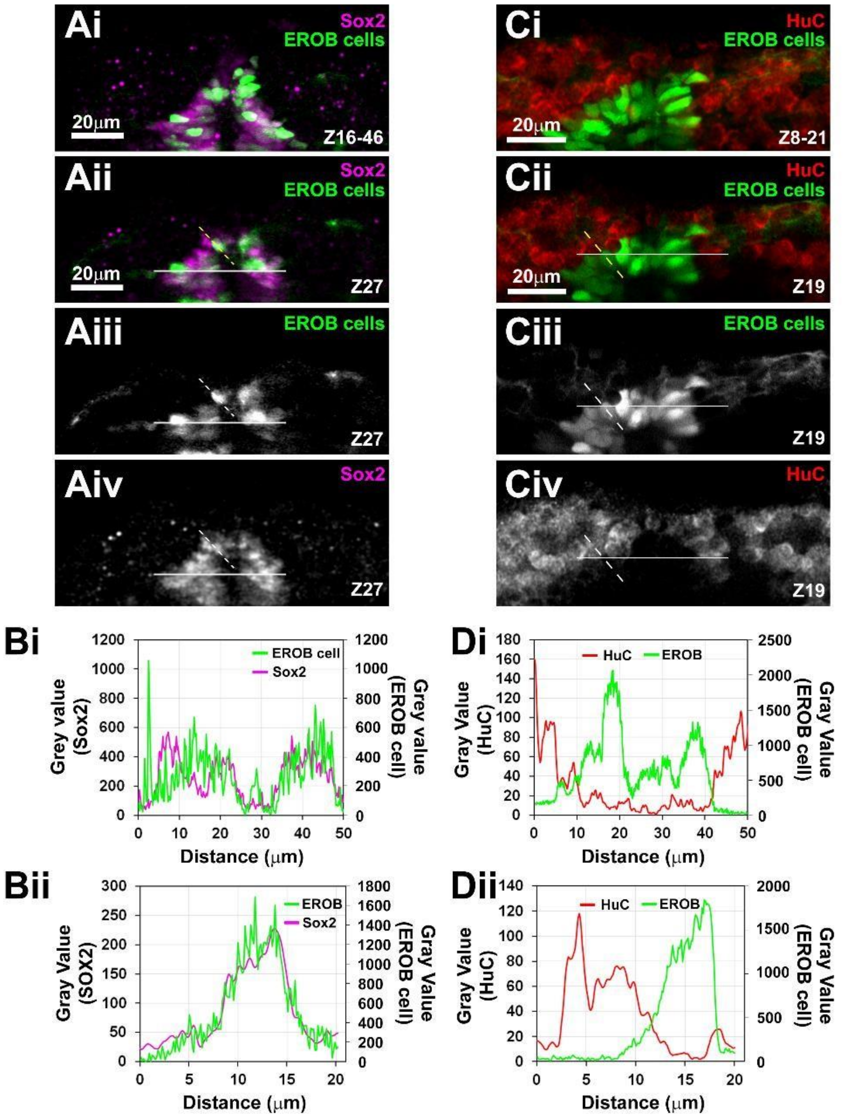

Fig. S1. EROB cells are Sox2 positive progenitors but not neurons. (Ai-iv) Confocal z projection image (Ai) and optical section images (1.5 μm step size) (Aii-Aiv) of EROB cells (GFP, green) and Sox 2 (red) in 4 dpf EE2-exposed ERE:GFP embryo. (Bi-ii) The intensity plot profiles show overlapping expressions of GFP (EROB cell nuclei) and Sox2, analysed along a white line (Bi, 50 μm) and a white dotted line (Bii, 20 μm) in Aii-iv. (Ci-iv) Confocal z-projection image (Ci) and optical section images (1.5 μm step size) (Cii-iv) of EROB cells (GFP, green) and HuC (Elavl3) (red) in 4 dpf EE2-exposed ERE:GFP embryo. (Di-ii) The intensity plot profiles show the segregated expressions of GFP (EROB cells) and HuC, analysed along a white line (Di, 50 μm) and a white dotted line (Dii, 20 μm) in Ci-iii.

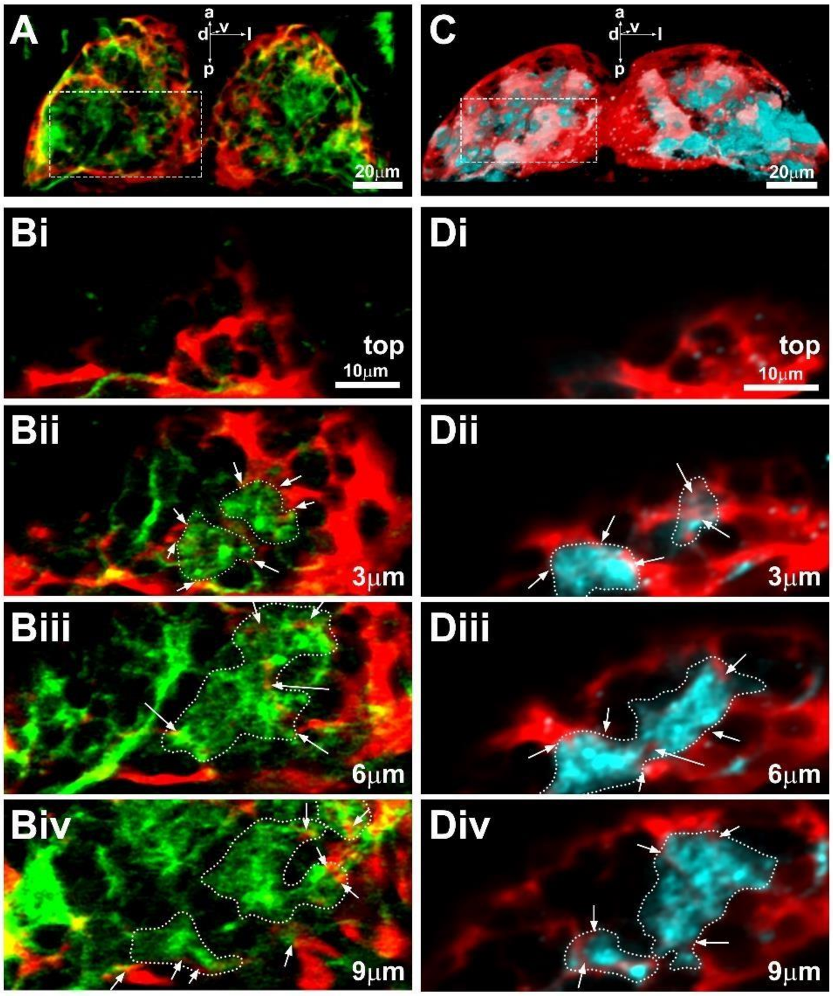

Fig. S2. The projections of EROB cells interact with olfactory sensory neurons at olfactory glomeruli. (A) A 3D image of EROB cells (red) and acetylated tubulin positive axonal projections (green) in the OB (dorsal view). d, dorsal; v, ventral; l, lateral; a, anterior; p, posterior. (Bi-iv) Sequential confocal optical section images (3 μm steps) of EROB cells (red) and acetylated tubulin^{+} axonal projections (green) in an mediodorsal area of the OB (indicated with white dotted rectangle in A) presenting along the dorso-ventral axis (top-bottom). White arrows, EROB cell-neuron contact sites in the OB glomeruli; white dotted lines, the outlines of glomeruli. (C) A 3D image of EROB cells (red) and olfactory glomeruli stained with SV2 antibody (cyan) (dorsal view). (Di-iv) Sequential confocal optical section images (3 μm steps) of EROB cells (red) and SV2+ olfactory glomeruli (cyan) in an mediodorsal area of the OB (indicated with white dotted rectangle in C) presenting along dorso-ventral axis (top-bottom). Arrows and lines indicate as above.

Fig. S3 Takesono etal.

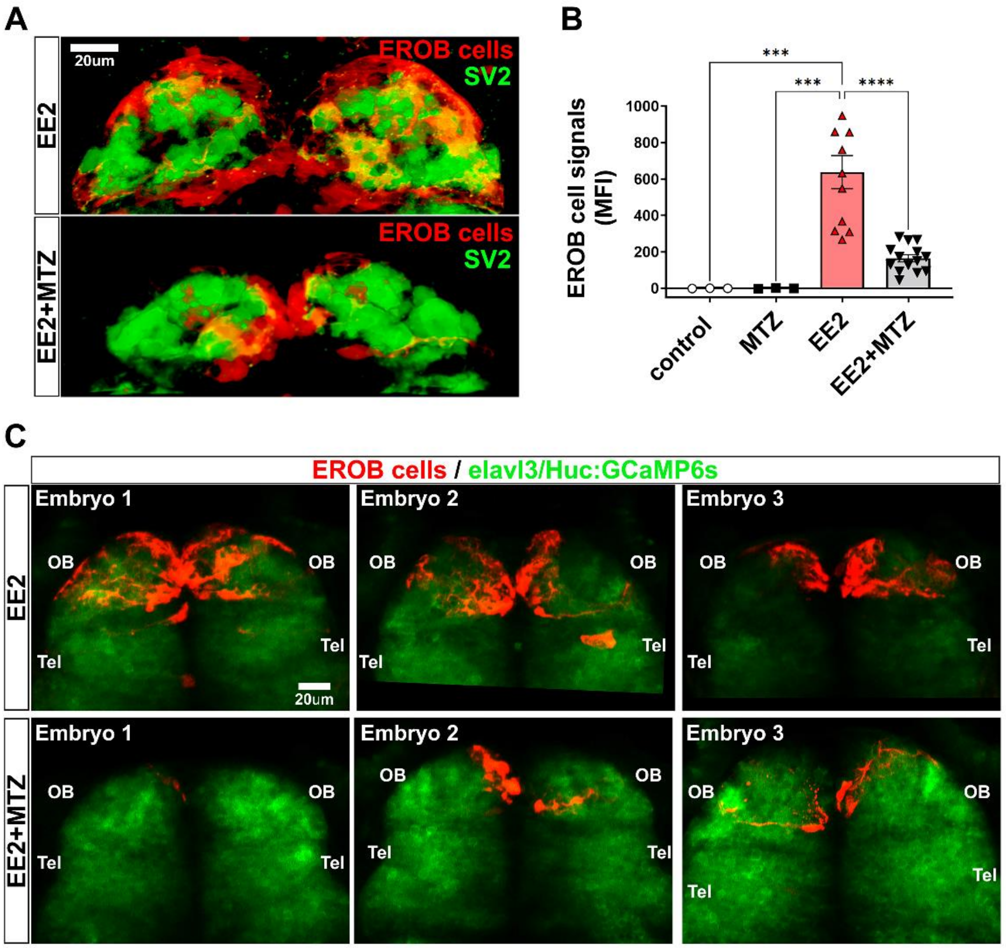

Fig. S3. EE2+MTZ treatment induces an efficient cell ablation of EROB cells.
(A) Representative 3D images of EE2 or EE2+MTZ treated 4 dpf ERE:mCherry embryos: EROB cells, red; SV2+ olfactory glomeruli, green. (B) Mean fluorescent intensity (MFI) of mCherry signals in the OB of control (N=3), MTZ (N=3), EE2 (N=12) and EE2+MTZ (N=12) treated 4 dpf ERE:mCherry embryos, which was obtained by subtracting the baseline MFI in other brain region in each fish. p*** < 0.001, p**** < 0.0001, ANOVA with Tukey post-hoc test. (C) Representative confocal z projection images of EROB cells (red) and time-averaged GCaMP6s signals (green) in 4 dpf EE2-exposed (top) or EE2+MTZ-exposed (bottom) 4 dpf Tg(*ERE:mCherry*) x Tg(*elavl3:GCaMP6s*) embryos. Images from three different embryos which were used for Fig. 6E-H are shown. Olfactory bulb, OB; Telencephalon, Tel.

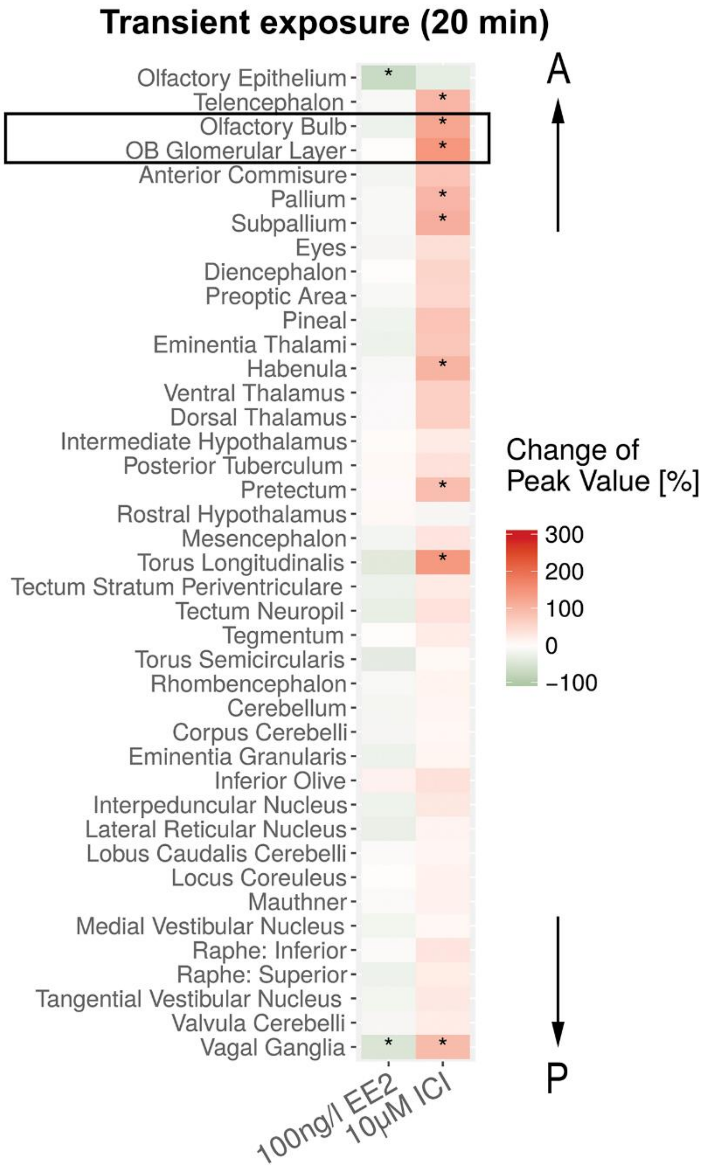

Fig. S4. Heat map displaying changes (%) of intrinsic neuronal activity in 41 different brain regions in 4 dpf elavl3:GCaMP6s embryos transiently exposed (for 20 minutes before LSM imaging) to EE2 (100 ng/L) or ICI (10 μM). p* < 0.05, the likelihood ratio test with Tukey's post-hoc test, N=8 each.

Development • Supplementary information

Fig. S5 Takesono etal.

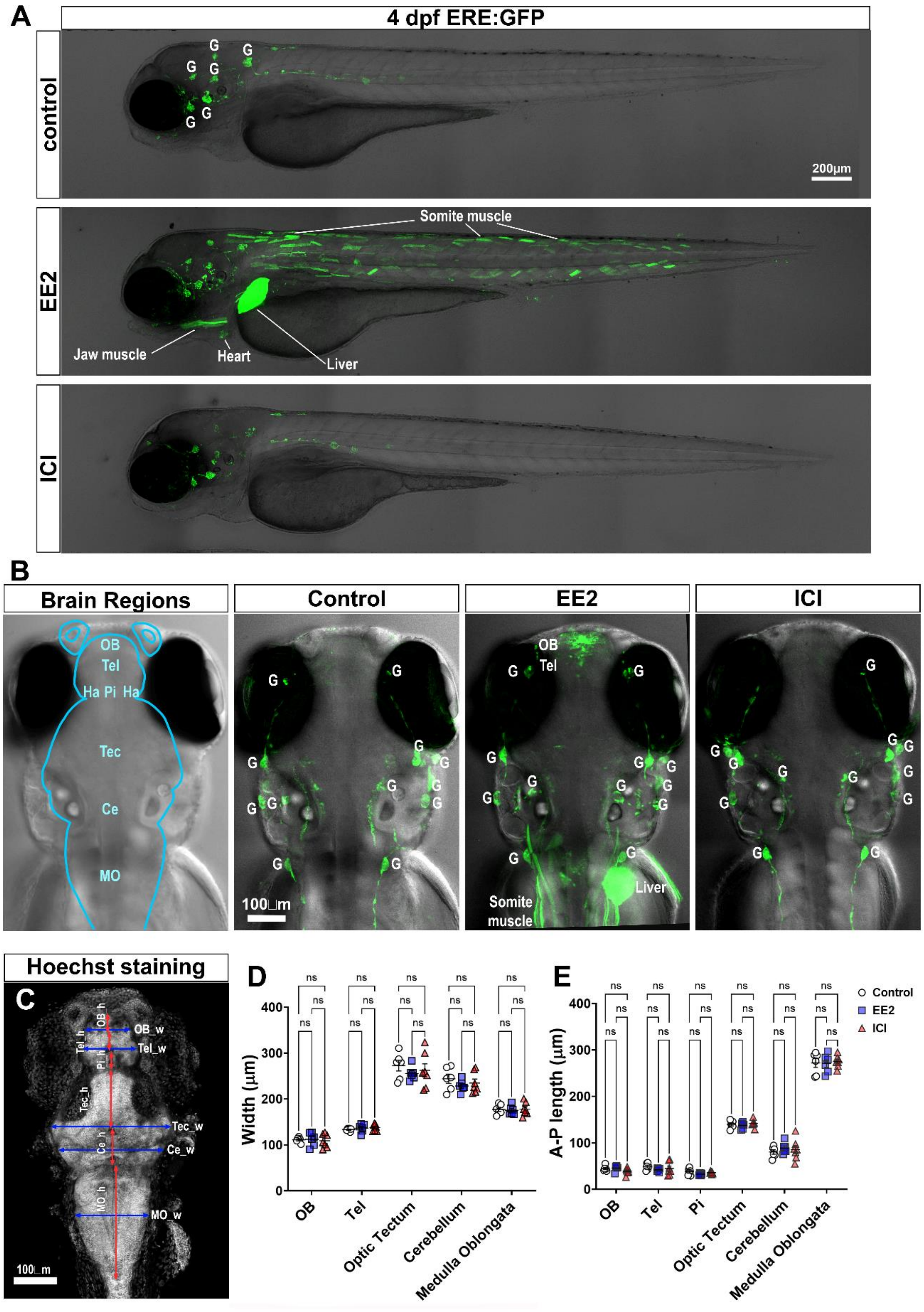

Development • Supplementary information

Fig. S5. EE2 or ICI exposure do not affect the global development of the body or the brain size in 4 dpf zebrafish embryos. (A) Whole body confocal z projection images of control (top), EE2– (middle) and ICI-exposed (bottom) 4 dpf ERE:GFP embryos. Note that ERE:GFP embryos show a consistent basal GFP expression in the ganglions (marked as "G"). EE2 exposure (from 1 to 96 hpf) induces GFP in the liver, heart and muscles, as previously reported. GFP expression in the brain is not easily visible in lateral orientation at this magnification (x10). ICI-exposure does not affect either the global development of the fish or the basal GFP expression. (B) Whole brain confocal z projection images of control, EE2- and ICI-exposed 4 dpf ERE:GFP embryos. Left end, a representative image showing the position of brain regions: olfactory bulb (OB); telencephalon, Tel; habenula, Ha; pineal, Pi; Optic tectum, Tec; cerebellum, Ce; medulla oblongata, MO. The outline of the brain is shown with a cyan line. EE2 exposure induces GFP expression predominantly in the OB (EROB cells)(the second image from right). (C-E) EE2 or ICI-exposure do not change the brain size. The width (D) and the A-P length (E) of brain regions were measured based on z projection images of Hoechst stained brain as indicated in C. Data were collected from control (white circles), EE2- (blue squares) and ICI-exposed (red triangles) 4 dpf ERE:GFP embryos. N=6-7. ANOVA with Tukey's test.

Development • Supplementary information

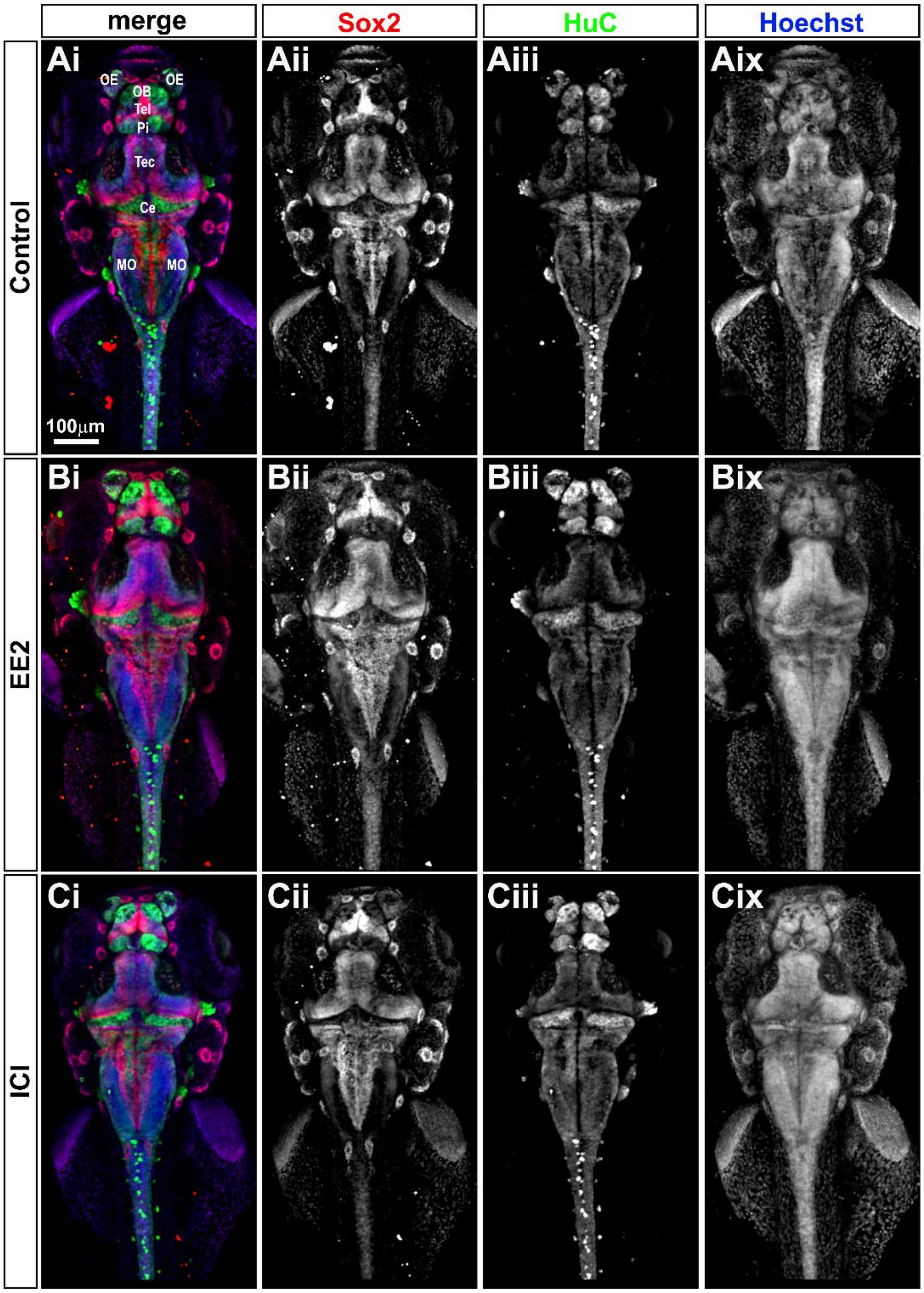

Fig. S6. EE2 or ICI exposure do not affect the expression domains of Sox2 or HuC in 4 dpf zebrafish embryos. (A) Whole mount confocal z projection images of control (top), EE2–(middle) and ICI-exposed (bottom) 4 dpf ERE:GFP embryos stained with neural stem cell marker, Sox2 (red), pan-neuronal marker, HuC (green) and Hoechst for nuclei (blue). Expression domains of these markers as well as the morphology of the whole brain are similar among the experimental groups (N=3).

Fig S7 Takesono et al.

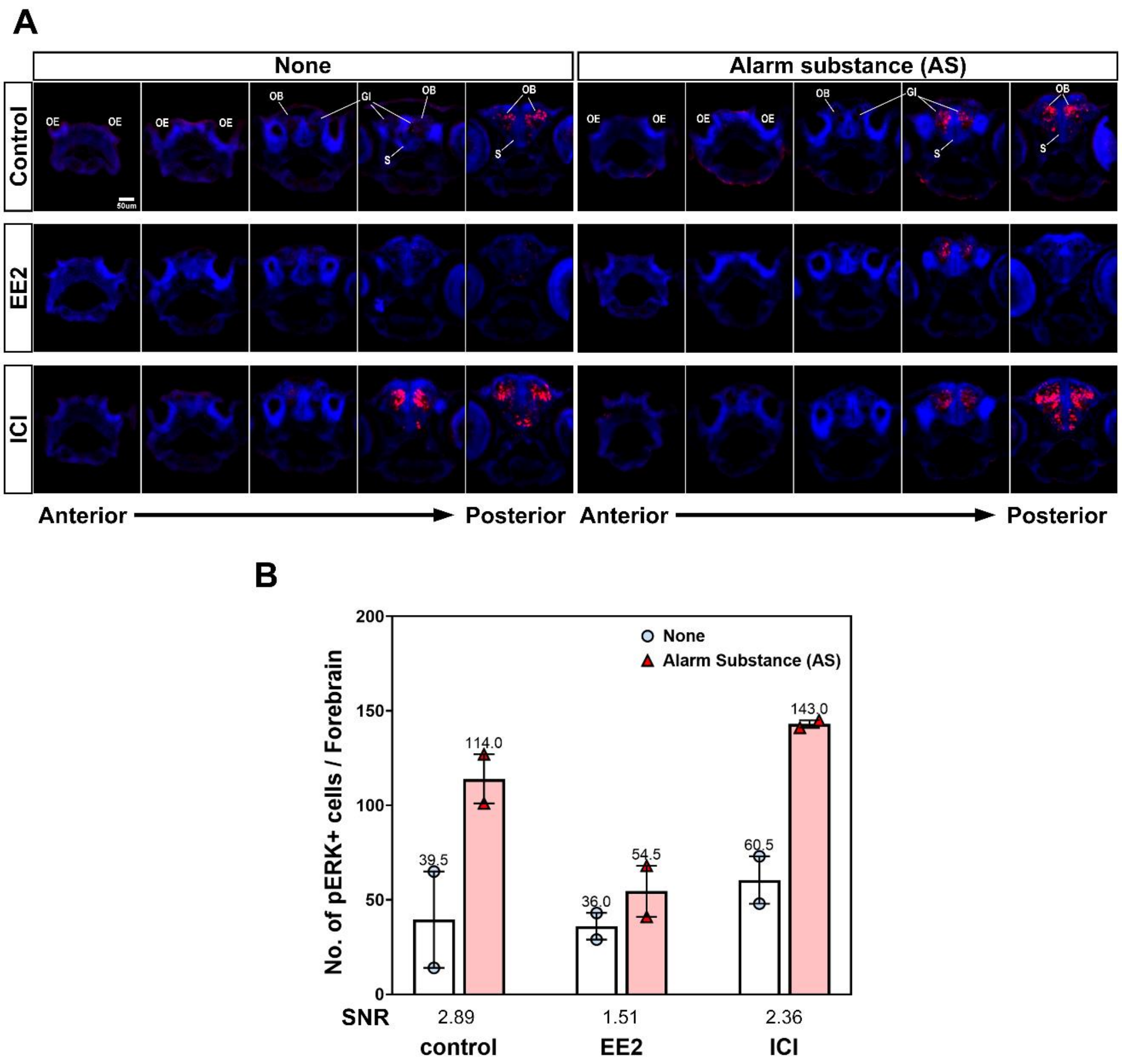

Fig. S7. Estrogens inhibit alarm substance-evoked neuronal activation. (A) Representative sequential forebrain transverse section images of pERK (red) and nuclei (blue)-stained none (left)- and cadavarine stimulated (right)- control, EE2, or ICI -treated 4 dpf ERE:mCherry larvae. Olfactory epithelia, OE; olfactory bulb, OB; OB glomeruli, Gl; subpallium, S; anterior-posterior axis. (B) pERK signals within ~50µm A-P volume of the forebrain in basal (white bar/pale blue dots)- or alarm substance (AS) stimulated (red bar/red triangles)- 4 dpf ERE:GFP larvae are shown (N=2). Mean values of each condition are shown above the bar. Signal/Noise Ratio (SNR) was calculated as “the mean of the odour-evoked pERK levels / the mean of the basal pERK levels” (shown below the x-axis).

References

Brion, F., Le Page, Y., Piccini, B., Cardoso, O., Tong, S. K., Chung, B. C. and Kah, O. (2012). Screening estrogenic activities of chemicals or mixtures in vivo using transgenic (cyp19a1b-GFP) zebrafish embryos. *PLoS One* **7**, e36069.

Gray, C., Loynes, C. A., Whyte, M. K., Crossman, D. C., Renshaw, S. A. and Chico, T. J. (2011). Simultaneous intravital imaging of macrophage and neutrophil behaviour during inflammation using a novel transgenic zebrafish. *Thromb Haemost* **105**, 811-819.

Green, J. M., Metz, J., Lee, O., Trznadel, M., Takesono, A., Brown, A. R., Owen, S. F., Kudoh, T. and Tyler, C. R. (2016). High-Content and Semi-Automated Quantification of Responses to Estrogenic Chemicals Using a Novel Translucent Transgenic Zebrafish. *Environ Sci Technol* **50**, 6536-6545.

Lee, O., Takesono, A., Tada, M., Tyler, C. R. and Kudoh, T. (2012). Biosensor zebrafish provide new insights into potential health effects of environmental estrogens. *Environ Health Perspect* **120**, 990-996.

Miyasaka, N., Arganda-Carreras, I., Wakisaka, N., Masuda, M., Sumbul, U., Seung, H. S. and Yoshihara, Y. (2014). Olfactory projectome in the zebrafish forebrain revealed by genetic single-neuron labelling. *Nat Commun* **5**, 3639.

Mueller, T., Wullimann, Mario F. (2015). *Atlas of early zebrafish brain development - a tool for molecular neurogene*: Elsevier BV.

Speedie, N. and Gerlai, R. (2008). Alarm substance induced behavioral responses in zebrafish (Danio rerio). *Behav Brain Res* **188**, 168-177.

Steinfeld, R., Herb, J. T., Sprengel, R., Schaefer, A. T. and Fukunaga, I. (2015). Divergent innervation of the olfactory bulb by distinct raphe nuclei. *J Comp Neurol* **523**, 805-813.

Winter, M. J., Windell, D., Metz, J., Matthews, P., Pinion, J., Brown, J. T., Hetheridge, M. J., Ball, J. S., Owen, S. F., Redfern, W. S., et al. (2017). 4-dimensional functional profiling in the convulsant-treated larval zebrafish brain. *Sci Rep* **7**, 6581.
