# Supplementary material for: Estrogens regulate early embryonic development of the olfactory sensory system via estrogen-responsive glia
Source: Development. 2022 Jan 13;149(1):dev199860. doi: 10.1242/dev.199860 (PMC8881738; doi:10.1242/dev.199860)
Supplement: Reviewer comments [file develop-149-199860_review_history.pdf]

Estrogens regulate early embryonic development of the olfactory sensory system via estrogen-responsive glia

Aya Takesono, Paula Schirrmacher, Aaron Scott, Jon M. Green, Okhyun Lee, Matthew J. Winter, Tetsuhiro Kudoh and Charles R. Tyler
DOI: 10.1242/dev.199860

Editor: Steve Wilson

Review timeline

Original submission:	4 June 2021
Editorial decision:	6 July 2021
First revision received:	18 October 2021
Editorial decision:	25 November 2021
Second revision received:	1 December 2021
Accepted:	2 December 2021

Original submission

First decision letter

MS ID#: DEVELOP/2021/199860

MS TITLE: Estrogen regulates early embryonic development of the olfactory sensory system via estrogen responsive glia

AUTHORS: Aya Takesono, Paula Schirrmacher, Aaron Scott, Jon M Green, Okhyun Lee, Matthew J Winter, Tetsuhiro Kudoh, and Charles R Tyler

I have now received all the referees' reports on the above manuscript, and have reached a decision. The referees' comments are appended below, or you can access them online: please go to BenchPress and click on the 'Manuscripts with Decisions' queue in the Author Area.

As you will see, the referees express considerable interest in your work, but two of them have some significant criticisms and suggestions for improving your manuscript. If you are able to address the concerns and revise the manuscript along the lines suggested, I will be happy receive a revised version of the manuscript. Please also note that Development will normally permit only one round of major revision.

We are aware that you may be experiencing disruption to the normal running of your lab that make experimental revisions challenging. If it would be helpful, we encourage you to contact us to discuss your revision in greater detail. Please send us a point-by-point response indicating where you are able to address concerns raised (either experimentally or by changes to the text) and where you will not be able to do so within the normal timeframe of a revision. We will then provide further guidance. Please also note that we are happy to extend revision timeframes as necessary.

Please attend to all of the reviewers' comments and ensure that you clearly highlight all changes made in the revised manuscript. Please avoid using 'Tracked changes' in Word files as these are lost in PDF conversion. I should be grateful if you would also provide a point-by-point response detailing how you have dealt with the points raised by the reviewers in the 'Response to Reviewers' box. If you do not agree with any of their criticisms or suggestions please explain clearly why this is so.

© 2022. Published by The Company of Biologists under the terms of the Creative Commons Attribution License (https://creativecommons.org/licenses/by/4.0/).

Reviewer 1

Advance summary and potential significance to field

In this extensive study Takesono et al investigated the role of estrogen in olfactory bulb development and function. They first revealed that a specific cell type in the olfactory bulbs, which they termed EROB cells, responded to estrogen via ER-mediated transcriptional activation. Later they further characterized EROB cells, and showed that these are radial glia-like cells, that innervates the surface of the olfactory bulbs and around the olfactory glomeruli. Next, they showed that treating the animals with EE2 and estrogen antagonist ICI alter the development of olfactory glomeruli, during which ICI clearly reduced the volume of olfactory glomeruli and likely olfactory bulbs. Genetic EROB cell ablation, generated a small effect similar to ICI treatment in a small subset of olfactory bulb glomeruli. Authors also showed that while ICI treatment reduces the number of gephyrin expressing inhibitory synapses. In the final section that authors argued that ICI treatment increase and EE2 treatment decrease the spontaneous calcium signals in the OB, and EE2 and ICI treatment abolishes cadaverine odor mediated avoidance.

In general, I think this an interesting and significant study with exciting potential to reveal the role of estrogen in brain development. The molecular characterizations of the estrogen related alterations are solid. However, the functional imaging and behavioral part needs better designed experiments and analysis to truly support authors claims (I will try to make few suggestions). Also, as of now, the story argues that EROB cells are really important for this phenomenon, but in fact the most solid perturbations are apparent with EE2 and ICI treatments, and EROB ablation with MTZ either have weak results or could not be performed due to some MTZ related limitations authors mentioned. I think this somehow questions the idea that if the mechanisms investigated here truly through EROB cells or we are looking at a broad developmental alteration of the entire brain, or even animals' body (I will also make few suggestions about this below).

Overall, the manuscript is well written and this is an interesting study. With some/small additional work/analysis, the authors might better support their specific claims. I highly recommend consideration of this manuscript for publication in Development, after considering some of the points I raised below.

Comments for the author

Major comments:
1) As I mentioned above while ICI and EE2 has strong effects, EROB manipulation is not that effective in perturbing the system. Is this perhaps because EROB are only a small part of the story, and ICI and EE2 manipulations have larger impact on larval fish development, by interfering with many other phenomena? For example, would ICI or EE2 treatment change the size of the brain or the entire animal? Would ICI or EE2 treatment alter the number inhibitory synapses in other parts of the brain (e.g. forebrain, optic tectum)?
2) As an extensions of this potential halted development due to estrogen pertubations, how much of this broad developmental alterations (that may be independent of EROB cells) would alter glomerular organization, neural excitability or even animal behavior ? None of these possibilities makes the impact of estrogen in this phenomenon uninteresting. However, the current data highlighting EROB function in all these results on brain excitability and animal behavior needs to be better supported, or authors can soften their claims on the specificity of these effects through EROB cells.
3) The functional imaging data is presented and analyzed rather inadequately, when compared to the standards of the field. I recommend the authors to better quantify neural excitability in EROB ablated and EE2/ICI treated animals.
4) Unfortunately, important odor responses are missing from calcium data. I guess this is partially due to difficulty in delivering odors in light sheet microscope. One potential, and rather simple experiment that could highlight the alterations in odor responses in EROB ablated and EE2/ICI treated animals is to perform pERK/tERK staining (See papers from Owen Randlett or Caroline Wee from Engert lab), to compare and investigate odor responses in these animals, perhaps using multiple odors ?. This will also be helpful to better evaluate the impact in other brain regions too.

© 2022. Published by The Company of Biologists under the terms of the Creative Commons Attribution License (https://creativecommons.org/licenses/by/4.0/).

5) The behavior data is also rather limited use, due to the use of a single odor. It is also not clear if ICI or EE2 alter overall development of locomotion ability, swim speed, or other swim characteristics in these animals. I also find it particularly pity that EORB ablation could not be done due to side effects of MTZ on cadaverine behaviors. I think this would have been absolutely an important experiment to claim the importance of EROB cells specifically for this phenomenon, beyond other possible actions of EE2 and ICI treatments in the brain or animals' body.

Minor comments
1) Please use scatter plots for better data visualization
2) Please mention animals age when presenting data. I saw that most experiments were done at 4dpf, but is it possible some experiments were done in different ages. I was a bit confused with animals age. Also in the abstract the developmental stage can be better clarified
3) Does ICI/EE2 treatments or EROB ablation have impact on the ORNs (odor responses, ciliation, number, density), or the number of inhibitory granule cells or excitatory mitral cells ?
4) The fact that ICI and EE2 generates similar behavioral perturbations and very different effect on calcium signals. This is rather confusing, can the authors expand on this, given all other observations? perhaps a scheme at the end might help to summarize and bind all results ?
5) "line 154: that EROB cells may act as cellular guides for directing the axonal growth of OSNs to specific glomeruli". Is this not a bit of over interpretation for the results? where is the evidence for axon guidance idea ?
6) Is the estrogen effect specific to inhibitory synapses? probably broader, given the overall developmental impact of EE2 and ICI treatments. As of now the data is presented in a way that communicates rather inhibitory synaptic effect for estrogen.

Reviewer 2

Advance summary and potential significance to field

There is little understanding on how estrogen exerts its effects in the embryonic brain before the period of sex differentiation. Takesono et al. studied the early developmental role of estrogen. Using a transgenic zebrafish line harboring an estrogen responsive reporter they have identified estrogen activity in olfactory bulb glial cells, which were named EROB. They next show that chronic exogenous treatment of estrogen starting from 1 hour post fertilization affects the development and function of on the olfactory bulb (OB).
They argue that these effects are mediated by a specific estrogen-induced transcriptional in their newly identified EROB glia. The concept of this manuscript as well as the proposed mechanism is new and intriguing and appropriate of publication in Development.

Comments for the author

I have some major concerns that need to be addressed by the authors. In particular, the specificity of the chemical and genetic perturbation as presented by the authors are in doubt due to the lack of characterization of other affected cells and possible indirect effects. I also had trouble reading the manuscript as many important technical details are not well described in the results section and I was compelled to go back and forth the methods and supplemental information in order to fully understand the experiments.

Specific major comments:
1) A general comment: Throughout the result section of the manuscript the authors are writing "details in Methods" so one has to go back and forth the results, methods and supplementary information to understand the methodology (see comment 6 for example). Authors should provide more details in the result sections so as to ease the reading .
2) Fig. 1: The authors show that the ERE:GFP reporter is specifically activated in the olfactory bulb after prolonged exposure ERE:GFP embryos to exogenous ethinylestradiol. As the author state "the ERE-TG models can detect ER-mediated transcriptional activities widely throughout the embryo without being affected by tissue specific enhancers or suppressors (Lee et al., 2012a)." This means that exogenous application of estrogen should light up the whole embryos. Also, why don't they see endogenous estrogen-induced transcriptional activity without estrogen exposure.

© 2022. Published by The Company of Biologists under the terms of the Creative Commons Attribution License (https://creativecommons.org/licenses/by/4.0/).

I understand that they are using the ERE:GFP line published by Green et al. 2016 and if this is the case, I see that as expected many cells are labelled in this reporter. The authors should provide a more detailed high resolution characterization of the GFP-labeled cells in this line with and without estradiol treatment.

3) The estrogen reporter was continuously activated in the olfactory bulb until 21 dpf. Was this due to endogenous response due to endogenous aromatase activity in EROB cells or have the authors kept exposing the fish to estradiol?

4) Figs 3-7: In all of these experiments the authors are chronically treating embryos with estrogen for 4 days starting at 1 hours post fertilization. As the authors state endocrine disrupting chemicals that mimic estrogens cause a wide range of adverse impacts on brain and other tissues development. So the prolonged estrogen treatment used throughout this study is likely to have a significant wide effects on embryonic development that might indirectly affect the OB, including EROB cells. The authors should address this major concern by providing evidence that the effects shown in all of these figures are specific (see my comments below).

5) Fig. 3: This experiment examine whether estrogen signaling is required for EROB cell projections however, it doesn't well support the authors claim. They exposed the embryos to exogenous estrogen for three days (1hpf-72hpf) and thereafter two days washout and then compared this so called 'control' embryos exposed to estrogen followed by estrogen receptor antagonist. The design of this experiment is puzzling. If they want to show estrogen receptor activity dependency they should compare untreated controls to embryos treated with estrogen receptor agonists and/or antagonist.

6) Fig. 4 and Fig. S3: Ablation of EROB cells affects OB glomeruli – Firstly, the description of the methodology in this section should be rewritten. The reader is forced to go through the Method section to understand that the MTZ-mediated ablation was performed with the Tg(ERE:Gal4ff; UAS:nfsBmCherry) line.

Secondly, in line with my comment number 4, the design of this experiment is not ideal to support the claim that they are specifically ablating EROB cells. Again, to my understanding, activation of an ERE:Gal4 driver following prolonged estradiol treatment is not specific to EROB cells as the transgene is expressed in all tissues. The authors do show in Fig. S3 that the activation (i.e. mCherry expression) is in a subset of OB cells showing a restricted field of view. However, the EE2+MTZ drug treatment is from 1 to 33 hpf so surely, other brain cells expressing the nitroreductase enzyme are being ablated. A more comprehensive characterization of the NTR-Cherry expressing cells before and after MTZ treatment should be provided as a measure of specificity.

7) Fig. 5: The analysis of OB glomeruli is based on spatial distribution and volume of SV-positive signal. I am not an expert but to me the definition of OB glomeruli locales seems arbitrary and I don't understand how one can make quantitative conclusions without the use of specific markers.

Reviewer 3

Advance summary and potential significance to field

This is a very thorough and carefully conducted study that explores the impact of estrogens on olfactory bulb development in the zebra fish model. The major advance is a detailed characterization of how a steroid hormone normally associated with either adult female reproductive function or the masculinization of the male mammalian brain following aromatization of testicular androgens, is a key component of brain development independent of sex and prior to the period for sexual differentiation of the brain. The impact of estrogens in this brain region is broad but coordinated, effecting projections, cell genesis and cell excitability and possibly involving a positive feedback loop with Cyp19a1b to locally control steroid synthesis and response.

Comments for the author

My recommendations are mostly minor

1) The authors are aware the "estrogen" refers to a class of compounds, one of which is estradiol, but the terminology is not used correctly. For instance, the title should either read "Estrogens regulate early..." or "Estradiol regulates early..." and so on throughout the manuscript.

© 2022. Published by The Company of Biologists under the terms of the Creative Commons Attribution License (https://creativecommons.org/licenses/by/4.0/).

2) The authors refer to "sex differentiation" but it is unclear to those not familiar with zebra fish if they are saying this means all the embryos are uni-sex at this point or if they are making a reference to the process of sexual differentiation of the brain in mammals.
3) the agonist and antagonists used have affinity for both ER-alpha and ER-beta.
It would be useful for the authors to discuss if there are analogous receptors in the zebra fish and if they think the effects they are seeing are due to one or the other receptor type.

First revision

Author response to reviewers' comments

Reviewer 1 Advance Summary and Potential Significance to Field:
In this extensive study Takesono et al investigated the role of estrogen in olfactory bulb development and function. They first revealed that a specific cell type in the olfactory bulbs, which they termed EROB cells, responded to estrogen via ER-mediated transcriptional activation. Later they further characterized EROB cells, and showed that these are radial glia-like cells, that innervates the surface of the olfactory bulbs and around the olfactory glomeruli. Next, they showed that treating the animals with EE2 and estrogen antagonist ICI alter the development of olfactory glomeruli, during which ICI clearly reduced the volume of olfactory glomeruli and likely olfactory bulbs. Genetic EROB cell ablation, generated a small effect similar to ICI treatment in a small subset of olfactory bulb glomeruli. Authors also showed that while ICI treatment reduces the number of gephyrin expressing inhibitory synapses. In the final section that authors argued that ICI treatment increase and EE2 treatment decrease the spontaneous calcium signals in the OB, and EE2 and ICI treatment abolishes cadaverine odor mediated avoidance.

In general, I think this an interesting and significant study with exciting potential to reveal the role of estrogen in brain development. The molecular characterizations of the estrogen related alterations are solid. However, the functional imaging and behavioral part needs better designed experiments and analysis to truly support authors claims (I will try to make few suggestions). Also, as of now, the story argues that EROB cells are really important for this phenomenon, but in fact the most solid perturbations are apparent with EE2 and ICI treatments, and EROB ablation with MTZ either have weak results or could not be performed due to some MTZ related limitations authors mentioned. I think this somehow questions the idea that if the mechanisms investigated here truly through EROB cells or we are looking at a broad developmental alteration of the entire brain, or even animals’ body (I will also make few suggestions about this below).

Overall, the manuscript is well written and this is an interesting study. With some/small additional work/analysis, the authors might better support their specific claims. I highly recommend consideration of this manuscript for publication in Development, after considering some of the points I raised below.

We thank this reviewer for both their encouraging response and constructive criticisms that we address in full below. On the requests and recommendations of this reviewer we have undertaken further experimentation to provide further new imaging and data and some additional analyses to further evidence our claims.

Reviewer 1 Comments for the Author:
Major comments:
1) As I mentioned above while ICI and EE2 has strong effects, EROB manipulation is not that effective in perturbing the system. Is this perhaps because EROB are only a small part of the story, and ICI and EE2 manipulations have larger impact on larval fish development, by interfering with many other phenomena? For example, would ICI or EE2 treatment change the size of the brain or the entire animal? Would ICI or EE2 treatment alter the number inhibitory synapses in other parts of the brain (e.g. forebrain, optic tectum)?

Answer to Q1: This is a very fair question. To address this question, through the use of Hoechst staining, we have measured the sizes of several different major brain regions (i.e. OB,

© 2022. Published by The Company of Biologists under the terms of the Creative Commons Attribution License (https://creativecommons.org/licenses/by/4.0/).

Telencephalon, Habenula/Pineal, Optic Tectum, Cerebellum and Medulla Oblongata) of 4 dpf zebrafish to confirm that EE2 or ICI treatment do not significantly alter their sizes compared with controls (shown in a new Fig. S3C-E). Furthermore, we also confirmed that EE2 or ICI treatment do not change the global development of the fish (shown in new Fig. S3A). We also show that the expression domains of two different early neural cell markers (HuC and SOX2) remain unchanged in both EE2 and ICI-treated animals (Fig. S4). Together with the data shown in this paper, these supplemental data further evidence that the effects of EE2 or ICI on the brain are likely to be specific to the OB development/function during early embryo development, and not globally affecting the entire brain development or development of the fish.

We agree with the comment that examining the effects of EE2 or ICI on the number of inhibitory synapses in other parts of the brain would valuable question to be address, but such further details are beyond the scope of this paper and an area for future study.

2) As an extensions of this potential halted development due to estrogen pertubations, how much of this broad developmental alterations (that may be independent of EROB cells) would alter glomerular organization, neural excitability or even animal behavior ? None of these possibilities makes the impact of estrogen in this phenomenon uninteresting. However, the current data highlighting EROB function in all these results on brain excitability and animal behavior needs to be better supported, or authors can soften their claims on the specificity of these effects through EROB cells.

Answer to Q2: In the original MS, we showed that phenotypes induced through estrogen perturbations by ICI, i.e. alterations in olfactory glomerular development (Fig. 4A-B) and OB-specific increase in the intrinsic neuronal activity (Fig. 6E-H), were recapitulated by EROB cell ablation (EE2+MTZ treatment). In our revised MS, we have added new data assessing olfaction-mediated (cadavarine and alarm substance) neuronal activity in the OB (pERK staining) to show that the basal and odour-evoked ERK phosphorylation was inhibited in EE2-treated fish (Fig. 7A-B, Fig. S7). Such EE2 effect on excitability were offset by EROB cell ablation (Fig. 7A-B, Fig. S7). In summary, in two different assay systems, GCaMP analyses (Fig. 6A-C and 6D-H) and pERK analyses (Fig. 7A- B), we consistently observed the inhibitory effects of EE2 on the intrinsic and/or olfaction-mediated neuronal activity while inhibition of estrogen pathway by ICI induced the inverse effects on neuronal activity (i.e. activation of GCaMP and pERK levels), and this was also the case for EE2 vs. EROB cell ablation. These data further support that the effects of EE2 on OB development and function are likely to be mediated by EROB cells.

Reviewer 1 rightly pointed, the phenotypes induced through EROB cell ablation on OB glomerular organisation or neuronal excitability are milder than those that were induced by ICI. We think this may be, at least in part, due to the fact that we needed to minimise the use of MTZ treatment for the period up to 33 hpf in order to minimize MTZ-induced cytotoxicity in other estrogen responsive cells/tissues, thus the reason why we could achieve a partial EROB cell ablation only (i.e. Fig. S5A-B). This idea is now explained in the text (lines 326-327).

In addition, we have also added a paragraph to discuss about other estrogen responsive brain cells which have previously been reported by the use of other estrogen biosensor zebrafish embryos which were exposed to exogenous estrogens (Brion et al., 2012; Gorelick and Halpern, 2011) in lines 316-320. To clarify whether the effects of estrogen on OB development/function solely depend on EROB cells, more complete ablation of EROB cell at a single cell level would be required and this idea is explained in the text (lines 327-331).

3) The functional imaging data is presented and analyzed rather inadequately, when compared to the standards of the field. I recommend the authors to better quantify neural excitability in EROB ablated and EE2/ICI treated animals.

Answer to Q3: The comment made here by this reviewer questions the manner in which we have quantified neural activity although no specifics guidance was provided on how they would improve this. To address this concern, in our revised MS, we have now presented the confocal GCaMP data as dF/F(%): We obtained the reference intensity level (baseline fluorescence F) for each fish corresponding to basal activity, of which signals from volumetric time series over ~ 2 min remain consistently lower than the signals in OB glomeruli. We found no significant difference in F between

© 2022. Published by The Company of Biologists under the terms of the Creative Commons Attribution License (https://creativecommons.org/licenses/by/4.0/).

the treatment conditions (shown in Fig. 6E). Functional signals were extracted from the volumetric time series and shown as dF/F(%) = % of the change in the fluorescence (delta F) normalised to the baseline fluorescence (F) for each fish. We found that there was no significant time-window deviation in dF/F (%) in all treatment conditions (Fig 6H).

LSM GCaMP analyses were conducted as described in our previous papers (Winter et al., 2021; Winter et al., 2017). With this, we found that the alterations of estrogen signalling by EE2 or ICI specifically affect the neuronal activity in the OB out of the entire brain regions (Fig 6A-C).

In order to achieve EROB cell ablation, we needed to use heterozygous [elavl3/HuC:GCaMP6s] x [ERE_nfsb:mCherry], which is the reason why the baseline elavl3/HuC:GCaMP6s signalling was much reduced (~70%). With this lower baseline, we were unable to perform quantitative GCaMP imaging of heterozygous [elavl3/HuC:GCaMP6s] x [ERE_nfsb:mCherry] embryo using our light-sheet microscopy system and to improve the detection sensitivity, we used Zeiss 880 airyscan in fast mode acquisition.

Through both methods, we have confirmed that EE2 inhibits the intrinsic neuronal activity in the OB, and ICI and EROB ablation induced the inverse of these effects.

4) Unfortunately, important odour responses are missing from calcium data. I guess this is partially due to difficulty in delivering odors in light sheet microscope. One potential, and rather simple experiment that could highlight the alterations in odor responses in EROB ablated and EE2/ICI treated animals is to perform pERK/tERK staining (See papers from Owen Randlett or Caroline Wee from Engert lab), to compare and investigate odor responses in these animals, perhaps using multiple odors ?. This will also be helpful to better evaluate the impact in other brain regions too.

Answer to Q4: As requested, we have now added pERK staining data, which indicates olfaction-mediated neuronal activation (increase in pERK signals, Fig. 7A-B). We confirmed that the effects of EE2, ICI or EROB cell ablation on neuronal activity are consistent in both live Ca^{2+} imaging and the pERK analyses (Fig. 6 vs Fig. 7A- B). We also added pERK staining data using alarm substance (AS) as another odourant cue (Fig. S7). The effects of EE2 or ICI on neuronal activity are consistent in both the cadavarine and AS-induced pERK responses.

In this paper, our focus is on the roles of estrogen signalling in the OB in the forebrain and therefore we do not include pERK data for the other posterior brain regions.

5) The behavior data is also rather limited use, due to the use of a single odor. It is also not clear if ICI or EE2 alter overall development of locomotion ability, swim speed, or other swim characteristics in these animals. I also find it particularly pity that EORB ablation could not be done due to side effects of MTZ on cadaverine behaviors. I think this would have been absolutely an important experiment to claim the importance of EROB cells specifically for this phenomenon, beyond other possible actions of EE2 and ICI treatments in the brain or animals' body.
Possible re-analysis: Locomotion ability (but this can be seen in acclimation swimming behaviour), swim speed, turning frequency etc.

Unfortunately, we are not able to add further behaviour data due to our current limitation in the use of animal over 5 dpf in accordance with UK Home Office. As this Reviewer points out above, we showed there was no apparent effect on general locomotion ability in EE2 or ICI-treated animals (acclimation, Fig. 7C-D). Thus, we do provide the evidence that links defective avoidance behaviour in EE2 or ICI treated animals with alterations in odour sensing in these animals, rather than by general adverse effects of EE2 or ICI on larval mobility/movement.

Minor comments
1) Please use scatter plots for better data visualization

Answers to Q1": As requested, we have accordingly changed the format of data of Fig. 3D-F, 4B, 6C, 6E-G and 7B and 7D to scatter plots.

© 2022. Published by The Company of Biologists under the terms of the Creative Commons Attribution License (https://creativecommons.org/licenses/by/4.0/).

2) Please mention animals age when presenting data. I saw that most experiments were done at 4dpf, but is it possible some experiments were done in different ages. I was a bit confused with animals age. Also in the abstract the developmental stage can be better clarified

Answers to Q2": We now include the stage of animal in the abstract. All data were obtained by using 4 dpf, zebraish larvae except for the data on EROB cell ontogeny (32 hpf upto 21 dpf), Fig. 3 and pERK staining (120hpf(5dpf)) and the behaviour assay (using 9-11 dpf). The ages of these animal are shown in figure legends as well as in Materials and Methods section.

3) Does ICI/EE2 treatments or EROB ablation have impact on the ORNs (odor responses, ciliation, number, density), or the number of inhibitory granule cells or excitatory mitral cells ?

Answers to Q3": We show that EROB cells are localised at the outer most layer of the OB and are adjacent to OSN (ORNs) layer (Fig. 2C-D, Fig.S2) and closely interact with OSNs at the OB glomeruli (Fig. 2E). We also show that ICI treatment or EROB cell ablation affect axonal extension of the OSNs (Fig. 4A-B). We further illustrate in Fig 7A-B that odour-mediated neuronal activation (assessed by pERK staining) was inhibited by EE2, while ICI or EROB cell ablation induced the opposite effects. These anatomical, developmental and functional evidence strongly suggest that OSNs are a major target of estrogen signalling pathway during brain development.

However, currently we do not know whether the effects of estrogen signalling pathway on OB development and function(s) are solely depending on the impacted OSNs or also mediated through the effects on other types of neurons in the OB, such as inhibitory interneurons and/or mitral cells. To further determine the specificity of estrogen functions in a specific type of neurons, detailed experiments using neuron type specific TG lines (i.e. TG lines for Olfactory Marker Protein (OMP) and TPRC2 for OSNs, lh2a for mitral cells and dlx4/6 for interneurons, and GCaMP lines for those specific neurons) would be necessary. The amount of work to do this is considerable and is beyond the scope of the current manuscript. We have however,now inserted a sentence on this issue - inserted in lines 346-349.

4) The fact that ICI and EE2 generates similar behavioral perturbations and very different effect on calcium signals. This is rather confusing, can the authors expand on this, given all other observations? perhaps a scheme at the end might help to summarize and bind all results ?

Answers to Q4": We have proposed a possible explanation for this in Page 13, line 372-375.

5)"line 154: that EROB cells may act as cellular guides for directing the axonal growth of OSNs to specific glomeruli". Is this not a bit of over interpretation for the results? where is the evidence for axon guidance idea ?

Answers to Q5: Our statement in line 154 in the previous MS was based on our descriptive evidence: 1) EROB cells and OSNs are closely localised in the OB and tightly interacting with each other to form spherical glomerular neuropils (Fig. 2), and 2) ICI or EROB cell ablation caused miss-localisation of olfactory glomeruli (Fig. 4A). Thus, we believe we provide good evidence that EROB cells may act as "cellular scaffold (previously described "cellular guide") for olfactory glomerular development. This concept is supported by previous descriptive analyses also in the rat embryonic brain where the termini of embryonic radial glia cells (RGCs) have been shown to intermingle with OSN axons to form the foundation of OB glomeruli, protoglomeruli, in the OB layer (Bailey et al., 1999; Ramon-Cueto and Valverde, 1995; Valverde et al., 1992) (lines 333-336).

The reviewer makes a fair point here, however, and we have change the language on this point. On original line 154 (now line 155 in the revised MS) we have changed the text from "cellular guide" to read "cellular scaffold". We hope that this better illustrates the possible role of EROB cells as a structural support for OB glomerular development, rather than as cells releasing axonal guidance cues etc. We also "to guild the extension of OSN projections into olfactory glomeruli and" in lines 378-379, respectively.

6)Is the estrogen effect specific to inhibitory synapses? probably broader, given the overall developmental impact of EE2 and ICI treatments. As of now the data is presented in a way that communicates rather inhibitory synaptic effect for estrogen.

© 2022. Published by The Company of Biologists under the terms of the Creative Commons Attribution License (https://creativecommons.org/licenses/by/4.0/).

We think this possibility should indeed be addressed in future studies and we have added text to discuss about this possibility in lines 346-349.

Reviewer 2 Advance Summary and Potential Significance to Field:
There is little understanding on how estrogen exerts its effects in the embryonic brain before the period of sex differentiation. Takesono et al. studied the early developmental role of estrogen. Using a transgenic zebrafish line harboring an estrogen responsive reporter they have identified estrogen activity in olfactory bulb glial cells, which were named EROB. They next show that chronic exogenous treatment of estrogen starting from 1 hour post fertilization affects the development and function of on the olfactory bulb (OB).
They argue that these effects are mediated by a specific estrogen-induced transcriptional in their newly identified EROB glia. The concept of this manuscript as well as the proposed mechanism is new and intriguing and appropriate of publication in Development.

Reviewer 2 Comments for the Author:
I have some major concerns that need to be addressed by the authors. In particular, the specificity of the chemical and genetic perturbation as presented by the authors are in doubt due to the lack of characterization of other affected cells and possible indirect effects. I also had trouble reading the manuscript as many important technical details are not well described in the results section and I was compelled to go back and forth the methods and supplemental information in order to fully understand the experiments.

We thank this reviewer for both their response and constructive criticisms, that we address in full below. In particular we address their concerns about the specificity of the induced effects - through the addition of more data, analyses, and explanatory text. We have also sought to incorporate more of the technical details where they are needed to help with the flow of the manuscript.

Specific major comments:

1) A general comment: Throughout the result section of the manuscript the authors are writing “details in Methods” so one has to go back and forth the results, methods and supplementary information to understand the methodology (see comment 6 for example). Authors should provide more details in the result sections so as to ease the reading.

Answers to Q1: With the wide range of methods employed and so many technical details it was not easy to embed all the methodological details to allow the reader to interpret the finding based on the reading the results section. We have nevertheless added technical details on methods where we thought they were most needed into the relevant sections to help with the reading of the paper. This includes at lines 118-119 for further explaining the EE2 exposure conditions for Fig 1E-J, and at line 180-182 for explaining EROB cell ablation. Due to the word limit, we needed however to put most of the detailed experimental procedures specifically in the Materials and Methods section.

2) Fig. 1: The authors show that the ERE:GFP reporter is specifically activated in the olfactory bulb after prolonged exposure ERE:GFP embryos to exogenous ethinylestradiol. As the author state “the ERE-TG models can detect ER-mediated transcriptional activities widely throughout the embryo without being affected by tissue specific enhancers or suppressors (Lee et al., 2012a).” This means that exogenous application of estrogen should light up the whole embryos. Also, why don’t they see endogenous estrogen- induced transcriptional activity without estrogen exposure.
I understand that they are using the ERE:GFP line published by Green et al. 2016 and if this is the case, I see that as expected many cells are labelled in this reporter. The authors should provide a more detailed high resolution characterization of the GFP-labeled cells in this line with and without estradiol treatment.

Answers to Q2: At the developmental stages that we used for this study, endogenous levels of estrogen activity in estrogen responsive tissues are not optically detectable in the ERE:GFP or ERE:mCherry lines. Importantly, an inability to detect endogenous estrogen responsive cells during early embryo/larval stages has been commonly seen in all other estrogen biosensor zebrafish lines established to date (e.g. Brion et al., 2012; Gorelick and Halpern, 2011). This is likely due to a

© 2022. Published by The Company of Biologists under the terms of the Creative Commons Attribution License (https://creativecommons.org/licenses/by/4.0/).

combination of the very low levels of endogenous estrogen circulating in embryo/larval stages and the detection sensitivity limits of the in ERE:TG lines. Thus, we need to expose the embryos to exogenous estrogen in order to visualise the estrogen responsive cells in ERE:TG embryos. We have now described the need to do this in the discussion of the revised MS (lines 286-292). Estrogen-responding cells in other tissues/organs and estrogenic chemical dependent tissue specificity in our ERE:TG models have already been characterised in our previous work (Green et al., 2016; Lee et al., 2012a; Lee et al., 2012b; Moreman et al. 2017). These transgenic lines allow for the detection of endogenous estrogen activity in older female fish >1.5 month (i.e. in the gonad, liver etc., unpublished) with a detection limit in ERE:GFP originally around 1 ng/L of EE2 (eq. 3.4×10^{-12}M)(Lee et al., 2012a).

We have added whole body confocal images of ERE:GFP embryos to our manuscript (Fig. S3A, lateral view of a whole body; Fig. S3B, whole brain view) with or without EE2 or ICI. ERE:GFP embryos possess a high signal-to- noise sensitivity with only a minor and consistent background expression in cranial and lateral ganglia (Fig. S3A). As we have stated in lines 290-292 and 314-316 of our revised MS, EE2 exposure to ERE:GFP embryo induces GFP (ER-mediated transcriptional activation) in the liver, muscle, muscles, heart (Fig. S3A) and in the forebrain, predominantly in the OB (Fig. S3B), corresponding to a wider roles of estrogen in the development and physiology of these target tissues/organs. However, importantly we have confirmed that the duration and exposure concentrations of EE2 or ICI treatment we used for this study did not change any of the following; global fish embryo development (Fig. S3A), whole brain regional specification and sizes (Fig. S3B), early neural cell marker expression pattern (Fig. S4) or aspects of fitness measures (baseline swimming capability, behaviour in acclimation: Fig. 7C-D). Based on these data, we believe that in all of our experimental conditions the impacts of estrogen activation (EE2) or perturbation (by ICI or EROB cell ablation) on global embryo development are minimal (at least at the level of our optical detection) and are specific to OB development and function.

3) The estrogen reporter was continuously activated in the olfactory bulb until 21 dpf. Was this due to endogenous response due to endogenous aromatase activity in EROB cells or have the authors kept exposing the fish to estradiol?

Answers to Q3: Fish were exposed to EE2 up to 4 days before the indicated developmental stages in order to visualise EROB cells (described in Materials and Methods). We have added a line to clarify this point in the revised MS (see lines 118-119 and line 456-457).

4) Figs 3-7: In all of these experiments the authors are chronically treating embryos with estrogen for 4 days starting at 1 hours post fertilization. As the authors state endocrine disrupting chemicals that mimic estrogens cause a wide range of adverse impacts on brain and other tissues development. So the prolonged estrogen treatment used throughout this study is likely to have a significant wide effects on embryonic development that might indirectly affect the OB, including EROB cells. The authors should address this major concern by providing evidence that the effects shown in all of these figures are specific (see my comments below).

Answer to Q4: We have addressed this point above (see answers to Q2) and relevant answers to this question are summarised in our reply to Q1 and Q2 from Reviewer 1.

Our ERE:TG lines were originally established by screening GFP or mCherry expression in EE2 exposed 4 dpf embryos: The embryos were exposed to 50-100ng/L EE2 from 0-3 dpf for screening the first 2-3 generations until we established homozygous fish. This means that the embryos exposed with EE2 at early stage were able to develop to healthy mature males and females. Using these founder generations, we have been able to maintain these lines for over 10 years without a serious problem in general health or reproduction. We thus think that the impacts of estrogenic chemicals (EDCs) on embryo/larvae development may be very specific to a particular estrogen responsive tissues/cells and to a specific developmental stage as seen in our study.

5) Fig. 3: This experiment examine whether estrogen signalling is required for EROB cell projections however, it doesn't well support the authors claim. They exposed the embryos to exogenous estrogen for three days (1hpf-72hpf) and thereafter two days washout and then compared this so called 'control' embryos exposed to estrogen followed by estrogen receptor

© 2022. Published by The Company of Biologists under the terms of the Creative Commons Attribution License (https://creativecommons.org/licenses/by/4.0/).

antagonist. The design of this experiment is puzzling. If they want to show estrogen receptor activity dependency they should compare untreated controls to embryos treated with estrogen receptor agonists and/or antagonist.

Answers to Q5: The reviewer makes a fair point here. We though needed to take this approach in order to visualise EROB cells, because it was not feasible to observe EROB cells in ERE:TG embryos without first exposing them to exogenous estrogen. In order to do the experiment suggested by the reviewer we would need to identify a specific marker for EROB cells expressed independently of the activation of estrogen signalling and to apply this gene/protein/promoter as a marker to visualise the EROB cells (by *in situ* staining, immunostaining or transgenic reporter technique). To develop such a marker a single cell transcriptome of EROB cells would be required. This would be an important next step in this work, as we have discussed in our MS (line 416-417), but it is beyond the focus of this MS.

In Fig. 3, we showed that application of ICI from 72 to120hpf disrupts the projection networks of EROB cells. These data indicate that estrogen activity is required for the establishment and/or maintenance of EROB cell networks in the OB.

To prevent potential confusion in our wording, “control” is now shown as “EE2 wash-off” and “ICI” as “subsequent ICI treatment” in line 163-165 and the legend for Fig. 3.

6) Fig. 4 and Fig. S3: Ablation of EROB cells affects OB glomeruli – Firstly, the description of the methodology in this section should be rewritten. The reader is forced to go through the Method section to understand that the MTZ-mediated ablation was performed with the Tg(ERE:Gal4ff; UAS:nfsBmCherry) line. Secondly, in line with my comment number 4, the design of this experiment is not ideal to support the claim that they are specifically ablating EROB cells. Again, to my understanding, activation of an ERE:Gal4 driver following prolonged estradiol treatment is not specific to EROB cells as the transgene is expressed in all tissues. The authors do show in Fig. S3 that the activation (i.e. mCherry expression) is in a subset of OB cells showing a restricted field of view. However, the EE2+MTZ drug treatment is from 1 to 33 hpf so surely, other brain cells expressing the nitroreductase enzyme are being ablated. A more comprehensive characterization of the NTR-Cherry expressing cells before and after MTZ treatment should be provided as a measure of specificity.

Answers to Q6: The timing of EE2+MTZ exposure (from 1 to 33 hpf) is the time-window when EROB cells just started developing (Fig.1E) and all other estrogen-responsive cells in the other tissues, such as the liver and the muscle, are still very limited in their development. We designed this exposure to minimise the possible indirect effects on OB development/function and/or global development caused by ablation of other cells expressing the nitroreductase enzyme under ERE promoter activity. We have added lines of text in the revised MS to explain this more clearly (lines 180-182) and added more explanations in “Methods” in lines 602-609.

We have also added a whole brain image of control, EE2 or ICI exposed 4 dpf ERE:GFP embryos in Fig S3B. This data shows estrogen-induced GFP expression is predominantly seen in the OB in the whole brain regions (Fig. S3B).

To further illustrate the specificity of EROB cells in the forebrain region and of MTZ effect on EROB cell ablation, we also included the images of a wider forebrain region from the experimental animals used in Fig. 6D-H (three individuals/ EE2 and EE2+MTZ conditions, Fig. S5B).
However, we appreciate that cell ablation system we conducted has some technical limitations. To further clarify whether the effects of estrogens on OB development/functions are solely dependent on EROB cells, a more complete cell ablation of the entire EROB cells (e.g. by single cell laser ablation) would be required. We have added a sentence to highlight this limitation of our cell ablation system and a possible approach to address this in subsequent work in lines 327-331.

7) Fig. 5: The analysis of OB glomeruli is based on spatial distribution and volume of SV-positive signal. I am not an expert but to me the definition of OB glomeruli locales seems arbitrary and I don’t understand how one can make quantitative conclusions without the use of specific markers.

© 2022. Published by The Company of Biologists under the terms of the Creative Commons Attribution License (https://creativecommons.org/licenses/by/4.0/).

Answers to Q7: The first point to make here is that topological location of each OB glomerulus is highly conserved across individual fish and the position of mdG2 is consistently localised at the most dorsal surface in the OB and can be used as a robust reference position. Secondly, it has been established that it is possible to distinguish each glomerulus by mapping its spatial distribution within the OB, without the use of a specific marker for a glomerulus (e.q: Miyasaka, N. et.al. Nature Commun. volume 5: 3639 (2014)). Furthermore, there are only a very limited number of such known markers for individual glomeruli. In fact, we have tried some of them (i.e. antibodies against G alpha o and S100 that are supposed to be specific to mdG2) and they did not allow us to reproduce specific staining for a single glomerulus. Details of how we analyse the volume of each glomerulus are described in Method section of Supplemental information.

Reviewer 3 Advance Summary and Potential Significance to Field:
This is a very thorough and carefully conducted study that explores the impact of estrogens on olfactory bulb development in the zebra fish model. The major advance is a detailed characterization of how a steroid hormone normally associated with either adult female reproductive function or the masculinization of the male mammalian brain following aromatization of testicular androgens, is a key component of brain development independent of sex and prior to the period for sexual differentiation of the brain. The impact of estrogens in this brain region is broad but coordinated, effecting projections, cell genesis and cell excitability and possibly involving a positive feedback loop with Cyp19a1b to locally control steroid synthesis and response.

Reviewer 3 Comments for the Author: My recommendations are mostly minor

1) The authors are aware the "estrogen" refers to a class of compounds, one of which is estradiol, but the terminology is not used correctly. For instance, the title should either read "Estrogens regulate early..." or "Estradiol regulates early..." and so on throughout the manuscript.

Answer to Q1: We have changed the text accordingly where “estrogen” means a class of compounds ”estrogens”. These changes are highlighted in yellow.

2) The authors refer to "sex differentiation" but it is unclear to those not familiar with zebra fish if they are saying this means all the embryos are uni-sex at this point or if they are making a reference to the process of sexual differentiation of the brain in mammals.

Answer to Q2: We have added lines 96-98 in the revised MS to clarify “gonadal” and “brain” sex differentiation in zebrafish.

3) the agonist and antagonists used have affinity for both ER-alpha and ER-beta. It would be useful for the authors to discuss if there are analogous receptors in the zebra fish and if they think the effects they are seeing are due to one or the other receptor type.

Answer to Q3: To date, the detailed analyses for isotype specific ER expression domains in zebrafish have technically been challenging due to the lack of good isotype specific antibodies. Most works so far have *used in situ* hybridization methods using the antisense probes or RT-PCR/QPCR for comparative quantitation of ER expression. ESR1 (ER¤ in mammals), ESR2a and ESR2b (two homologs of ER¤ in mammal) have been shown to broadly be expressed in the brain of 24 and 48 hpf zebrafish embryo: possibly ESR2a>ESR2b>>ESR1 (Tingaud-Sequeira et al., 2004). There is no information of ER isotypes in a specific brain region in the embryonic brain.

We have added to the MS at line 302-303 to clarify this point.

© 2022. Published by The Company of Biologists under the terms of the Creative Commons Attribution License (https://creativecommons.org/licenses/by/4.0/).

Second decision letter

MS ID#: DEVELOP/2021/199860

MS TITLE: Estrogens regulate early embryonic development of the olfactory sensory system via estrogen responsive glia

AUTHORS: Aya Takesono, Paula Schirrmacher, Aaron Scott, Jon M Green, Okhyun Lee, Matthew J Winter, Tetsuhiro Kudoh, and Charles R Tyler

I am happy to say that the referees are happy with your revisions and there is just one point for you to consider before we proceed to publication. The referees' comments are appended below, or you can access them online: please go to BenchPress and click on the 'Manuscripts with Decisions' queue in the Author Area.

Reviewer 1

Advance summary and potential significance to field

I already summarized this in my initial review.

Comments for the author

I thank for the authors for the additional data and analysis, which certainly better supported authors claim, especially with respect to altered odor responses in the olfactory bulbs.
Moreover, the authors makes valid argument about whey some of my suggestions better suits a future study.
Together with the additional the textual changes, all my comments are sufficiently addressed.
I only want to make a minor point about the calcium imaging data. It would be great to see the time courses of odor responses (DF/F) from the olfactory bulbs so that we can better visualize the alterations in these signals. But I leave this to the authors.
I do not have further comments.
I support the publication of this manuscript in Development

Reviewer 2

Advance summary and potential significance to field

The authors have address my comments to my satisfaction. The manuscript is greatly improved and I now support the acceptance of the manuscript to Development.

Comments for the author

The authors have address my comments to my satisfaction. The manuscript is greatly improved and I now support the acceptance of the manuscript to Development.

Second revision

Author response to reviewers' comments

Responses to the reviewers -Version 3

Reviewer 1 Comments for the Author:
I thank for the authors for the additional data and analysis, which certainly better supported authors claim, especially with respect to altered odor responses in the olfactory bulbs.

© 2022. Published by The Company of Biologists under the terms of the Creative Commons Attribution License (https://creativecommons.org/licenses/by/4.0/).

Moreover, the authors makes valid argument about whey some of my suggestions better suits a future study. Together with the additional the textual changes, all my comments are sufficiently addressed.

We thank reviewer 1 for his/her time to read through our revised manuscript and for supporting the publication of our manuscript in Development.

I only want to make a minor point about the calcium imaging data. It would be great to see the time courses of odor responses (DF/F) from the olfactory bulbs so that we can better visualize the alterations in these signals. But I leave this to the authors.

Our responses:
We believe that the reviewer 1 suggested to show extra data for demonstrating how estrogen alters an odourant- stimulated GCaMP signals in real-time. We do not have such data at this time. We have tried to get such data but we have not been able to do so because of some major experimental challenges. The main issue here is the challenge in the optimisation of live sample mouting both in a glass capillary for the lightsheet microscopy and in a glass-bottom dish for fast-mode airyscan. This would be needed to enable us to expose the fish to an odour at the location of the nostrils while simultaneously recording live calcium imaging. Such work would require considerable further modifications in our existing approaches and settings for the microscopes. Given these technical challenges, we are not able to provide the specific additional information requested by this reviewer (which was a suggestion and not an explicit requirement). We would propose to develop such an approach in our longer term work.
We have though now included odour-stimulated pERK staining in our revised manuscript which was also requested by reviewer 1.

I do not have further comments.

I support the publication of this manuscript in Development

Reviewer 2 Advance Summary and Potential Significance to Field:
The authors have address my comments to my satisfaction. The manuscript is greatly improved and I now support the acceptance of the manuscript to Development.

Reviewer 2 Comments for the Author:
The authors have address my comments to my satisfaction. The manuscript is greatly improved and I now support the acceptance of the manuscript to Development.

Our responses:
We thank reviewer 2 for his/her time to read through our revisions. We are pleased to know that the revised manuscript was now satisfactory and this reviewer supports the acceptance of our paper.

Third decision letter

MS ID#: DEVELOP/2021/199860

MS TITLE: Estrogens regulate early embryonic development of the olfactory sensory system via estrogen responsive glia

AUTHORS: Aya Takesono, Paula Schirrmacher, Aaron Scott, Jon M Green, Okhyun Lee, Matthew J Winter, Tetsuhiro Kudoh, and Charles R Tyler
ARTICLE TYPE: Research Article

I am happy to tell you that your manuscript has been accepted for publication in Development, pending our standard ethics checks.

© 2022. Published by The Company of Biologists under the terms of the Creative Commons Attribution License (https://creativecommons.org/licenses/by/4.0/).
